# Examining changes in personality following shamanic ceremonial use of ayahuasca

**DOI:** 10.1038/s41598-021-84746-0

**Published:** 2021-03-23

**Authors:** Brandon Weiss, Joshua D. Miller, Nathan T. Carter, W. Keith Campbell

**Affiliations:** University of Georgia, Athens, GA USA

**Keywords:** Psychology, Human behaviour, Pharmacology

## Abstract

The present study examines the association between the ceremonial use of ayahuasca—a decoction combining the *Banistereopsis caapi* vine and N,N-Dimethyltryptamine-containing plants—and changes in personality traits as conceived by the Five-Factor model (FFM). We also examine the degree to which demographic characteristics, baseline personality, and acute post-ayahuasca experiences affect personality change. Participants recruited from three ayahuasca healing and spiritual centers in South and Central America (N = 256) completed self-report measures of personality at three timepoints (Baseline, Post, 3-month Follow-up). Informant-report measures of the FFM were also obtained (N = 110). Linear mixed models were used to examine changes in personality and the moderation of those changes by covariates. The most pronounced change was a reduction in Neuroticism *dz*_self-reportT1–T2_ =  − 1.00; *dz*_self-reportT1–T3_ =  − .85; *dz*_informant-reportT1–T3_ =  − .62), reflected in self- and informant-report data. Moderation of personality change by baseline personality, acute experiences, and purgative experiences was also observed.

## Introduction

The revival of interest in psychedelic medicines has two primary strands. First, laboratory-based clinical research focusing on therapeutic applications of serotonergic psychedelic compounds (i.e., 5-HT_2A_ receptor agonists, e.g., psilocybin, LSD) has yielded some evidence of long-term changes in personality^1,2^ and positive mental health outcomes^1,3^. Second, a parallel interest in shamanic medicine using psychedelic compounds (e.g., huachuma, ayahuasca) has also emerged but has received considerably less research attention. The ceremonial use of plants and fungi containing 5-HT_2A_ receptor agonists may date back two thousand years, involving numerous early cultures including the Aztec^5,6^, Native American^96^, and Grecian^6^ cultures, though Grecian use of ergot remains speculative.

The purpose of this study is to examine personality change following the ceremonial use of ayahuasca, a decoction combining the woody vine *Banistereopsis caapi*^8^ (containing β-carboline monoamine oxidase inhibitors) and plants containing the 5-HT2A receptor agonist N,N-Dimethyltryptamine (DMT; e.g., the shrub *Psychotria viridis*^9^, the vine *Diplopterys cabrerana*). Personality change is important to understand because personality is related to a wide array of important outcomes (e.g., occupational success^10^; physical health and mortality^7^), and serves as the foundation for many models of psychopathology (e.g., HiTOP^97^). Because personality change may depend on other measurable factors, we also examined the degree to which predisposing factors, such as demographic characteristics and baseline personality, and experiential factors, such as non-ordinary states of affect and consciousness during ceremony, moderate personality changes.

### The importance of studying ayahuasca in the ceremonial versus laboratory setting

Ayahuasca is thought to have a long history of ceremonial use among indigenous peoples of Brazil and the Amazonian basin of South America^6,11^. Scholars differ on the precise timing of ayahuasca shamanism’s development, with some proposing that modern ceremonial practice emerged no more than 300 years ago in a Spanish missionary context^12,13^; whereas other scholars assert modern practices have been uninterrupted among indigenous groups for five thousand years^14^. Archeological evidence supporting the latter position is, however, limited^15^. Within the last 25 years, ayahuasca healing centers have become sources of alternative mental health treatment among Westerners, particularly those whose symptoms have shown recalcitrance to change using Western approaches. In view of a long period of development, ceremonial practices may be informative about key elements of psychedelic-assisted experience that potentiate positive psychological changes. Furthermore, exploring approaches to healing that lie outside the Western scientific tradition is congruent with recent calls to revise Western epistemological biases that circumscribe scientific understanding^16,17^. Ayahuasca ceremonies combine numerous elements which may play a role in whatever personality change is found, including communal/group formats, guiding elements (e.g., chanting of prayer during ceremony, use of adjunct plants such as tobacco, perfumated water), engagement with a shaman (e.g., *icaro* [medicine prayer] delivered by the shaman), and engagement with personal challenges (e.g., purgative aspects, emotional intensity, traumatic reexperiencing). Purging, occurring generally within the first 2 h of ceremony, is typically preceded by digestive discomfort, and accompanied by feelings of physical and emotional relief.

A further element that deserves consideration is the ontological framework within which shamanic traditions understand the therapeutic process. For Shipibo shamans, the ayahuasca decoction has a spirit which aids in the healing process and serves as a sensory amplifier that facilitates awareness of spirits, particularly those imparting “dirty,” “calcified,” or “diseased” energies (“mahua yoshin” in Shipibo language, translated as energies/spirits of the dead). These energies are understood as blockages to health and are identified by dark or muddy colors and/or other visionary signifiers. Shipibo shamans observe these spirits/energies at four putative levels of the human being: nete (the world of the individual), shinan (thoughts/beliefs/mental contents), winti (heart/emotions/desires), and yora (dense physical body; e.g., blood, mucus, bones, flesh). The shaman’s icaro—a prayer regarded to have originated from previously “dieted” traditional plants/spirits—is thought to open up “portals” that guide positive spirits (operating as “muses” or “doctors”) to extract and remove *mahua yoshin* from ceremony participants. For Shipibo shamans, health may be characterized by connection with oneself, community, and the larger world. As such, there are emotional, spiritual, and moral aspects to the Shipibo’s conception of optimal health.

The (“dieta”) process may be worth highlighting as well because it is outside Western medical and scientific training. Dieta involves spending a period of time in isolation consuming a plant teacher (e.g., chu-wasi, chiric-sanango, pinon blanco, tobacco, coca) and focusing on its psychospiritual effects/teachings. Ideally, the spirit of the plant reveals itself and becomes an ally of the student. The dieta has three functions: purificatory (purifying physical, emotional, and mental aspects of the person), cultivation of intuition (innate knowing through interconnection), and bestowing by the plant teacher of protection (correspondence with anonymous Shipibo-trained shaman). Many of the Shipibo shamanic concepts presented here may be reduced in translation given their broad ontological context.

### Psychedelic research as a window into personality change

To date, psychologists have observed a number of pathways by which personality changes, including normative development^18^, biological maturation^19^, genetic factors^20^, major life events^21,22^, new social and vocational roles^23,24^, commitment to new identities^25,26^, psychotherapy^27,28^, and self-motivation^29,30^. Although some evidence suggests that psychedelic compounds may offer an additional pathway, prospective studies in naturalistic and laboratory settings have yielded mixed evidence.

Prospective studies in controlled laboratory settings have shown heterogeneous effects that may depend in part on length of follow-up, sample size, conditions of administration, and experiences during the acute effects of the compounds. Changes in Five-Factor model (FFM^31^) Neuroticism (decreasing), Extraversion, Openness, Agreeableness, Conscientiousness (increasing), and Absorption^32^ have been reported across healthy^2,33–35^ and clinical^1^ samples. Furthermore, two naturalistic studies have observed adaptive personality changes—decreases in Harm Avoidance (i.e., characterized by worry^36^)^37,38^ and increases in Self-Directedness (i.e., adaptive capacity to achieve chosen goals)^38^, personality domains that largely map onto FFM domains of Neuroticism, Extraversion, and Conscientiousness. Nevertheless, meaningful changes in these domains have not always been observed^39–42^, and the emergence of stable changes may depend on inner experiences during the compound’s acute effects^1,2^, c.f.^41,42^.

Finally, five clinical studies, including two randomized placebo-controlled trials, have provided support for an antidepressant effect^43–46^. Given that many psychopathological symptoms are increasingly conceptualized as maladaptive variants of basic personality dimensions and share the same structure^13,47^, these results may be relevant to FFM Neuroticism and Extraversion.

In view of the empirical inconsistency in these findings and null results within placebo-controlled studies^41^, one must consider that observed changes in self-reported personality may be products of placebo, expectancy, and/or demand effects following particularly intense and compelling experiences^48^. Methodological limitations may also account for empirical inconsistency. First, existing studies tend to use small samples (mean *N* = 25) and thus have lower statistical power, yield less precise estimates, and may be less generalizable. Second, even consistent findings have not been sufficiently replicated, with just two prospective examinations of ayahuasca-induced personality change having been published^37,38^. Third, few studies have corroborated self-reported change with observations of informant-reported change, which could reduce the influence of placebo, demand, and expectancy effects among target participants. Fourth, the majority of studies did not employ a control group or condition, which can accompany validity threats including regression to the mean and the Hawthorne effect^50^. Finally, few studies have rigorously examined potential moderators (e.g., sample, design characteristics) which may provide the necessary conditions for change, or have been sufficiently well-powered to validly do so.

### Ayahuasca moderators: set and setting

Theoretical and empirical work have pointed to a number of predisposing and experiential factors as having potential to account for variability in the long-term effects of psychedelic compounds.

#### Predisposing factors

Predisposing factors roughly map onto popularly observed determinants of psychedelic experience, (mind)set and setting^50^, but also include individual differences such as baseline personality traits. Among previously examined predisposing factors, personality traits (e.g., Absorption, or one’s disposition toward total attentional engagement with one’s perceptual or ideational resources), affective states (e.g., emotional excitability), age, and experimental setting have shown associations with affective and mystical states during psychedelic experience^1,51^.

#### Experiential factors

A small literature has examined acute non-ordinary psychological states as potentiating factors in personality change^1,2,4,39,41,42,51–53^. Among the most popular targets of inquiry are states of mystical-type and intense emotional experiences based on work indicating convergence between psychedelic, religious, psychodynamic (i.e., involving confrontation with self, emotion, and conflict), and transpersonal (i.e., involving continuity between mental, physical, and metaphysical life) phenomenology^52–55^. Previous findings have indicated that non-ordinary states of unitive consciousness (i.e., feeling of being one with a larger whole), insightfulness (i.e., perceptions of encounter with ultimate reality), awe, and transcendence from time and space (collectively referred to as mystical-type experience)^56^ may potentiate change in FFM Openness^2^, c.f.^1,42,43^, Neuroticism^1^, and Extraversion^1,57^.

## Present study

The present study examined personality change in relation to the ceremonial use of ayahuasca in a sample of 256 participants using self- and informant-report measures of personality across three timepoints (i.e., Baseline, Post, 3-month Follow-up for self-report; Baseline, 3-month Follow-up for informant-report). Differences in self- and informant-report personality domain scores between timepoints were examined. In line with previous work^1,2^, self- and informant-report FFM Openness and Extraversion were hypothesized to increase, and FFM Neuroticism was hypothesized to decrease following initial measurement.

The second aim was to investigate factors that may affect the degree of personality change found in relation to psychedelic experience. Specifically, we examined the degree to which differences in FFM traits and facet scores between timepoints varied as a function of predisposing and experiential factors. In line with previous work^2^, mystical-type experience was hypothesized to contribute to a larger difference in FFM Openness between Baseline, on one hand, and Post and Follow-up, on the other. All hypotheses were preregistered using the Open Science Foundation web platform (https://osf.io/xk3ym).

## Results

### Examining personality change

**Figure 1:**
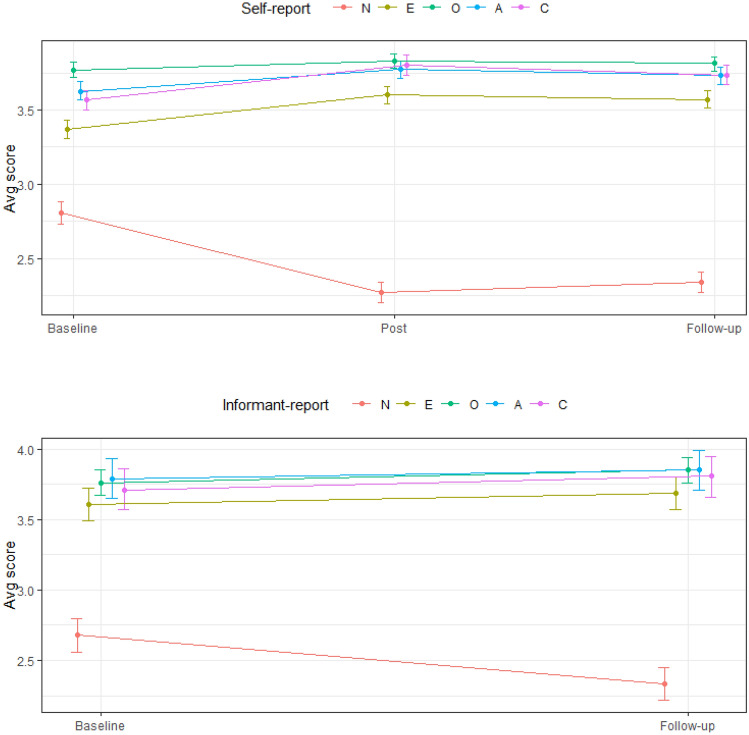
Line plots illustrating self-report and informant-report change in personality domains. Error bars represent 95% confidence intervals (for Neuroticism, Extraversion, Openness) and 99% confidence intervals (for Agreeableness, Conscientiousness).

**Figure 2:**
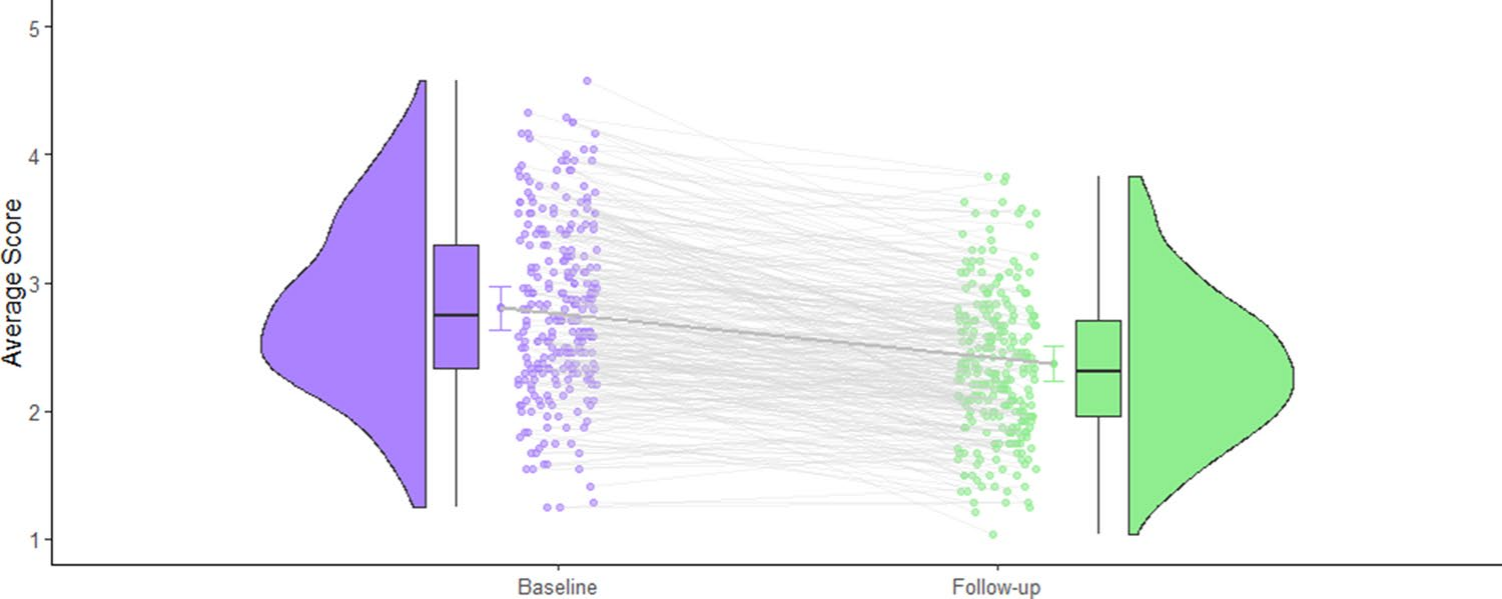
Box and Violin plots^98^ illustrating self-reported change in Neuroticism between Baseline and Follow-up.

With respect to self-report data, main effects of timepoint were observed on Neuroticism (F[2, 510] = 174.98, *p* < .0001, *mR*^2^ = .14), Extraversion (F[2, 510] = 58.39, *p* < .0001, *mR*^2^ = .04), Openness (F[2, 510] = 7.27, *p* = .001, *mR*^2^ = .004), Agreeableness (F[2, 510] = 40.82, *p* < .0001, *mR*^2^ = .03), and Conscientiousness (F[2, 510] = 70.18, *p* < .0001, *mR*^2^ = .05). Unstandardized (B) coefficients indicate mean differences between timepoints. *dz* indicates effect size change in personality scores in terms of the standard deviation of within-subject change scores (e.g., T2–T1). Cohen’s *d* (*ds*) represents effect size change in terms of the pooled standard deviation of personality scores at two timepoints. Post-hoc tests demonstrated that within a week following ayahuasca ceremony Neuroticism (B = − .53 95% CI [− .47, − .60], *dz* = − 1.00, *ds* = − .62) was significantly lower, and Extraversion (B = .23 95% CI [.18, .28], *dz* = .57, *ds* = .30), Openness (B = .06 95% CI [.03, .09], *dz* = .22, *ds* = .11), Agreeableness (B = .14 99% CI [.10, .19], *dz* = .52, *ds* = .27), and Conscientiousness (B = .23, 99% CI [.18, .29], *dz* = .70, *ds* = .38) were significantly higher. Three months following ayahuasca ceremony, Neuroticism (B = − .46 95% CI [− .40, − .53], *dz* = − .85, *ds* = − .53) remained significantly lower, and Extraversion (B = .20 95% CI [.15,.25], *dz* = .52, *ds* = .26), Openness (B = .05 95% CI [.01–.08], *dz* = .16, *ds* = .08), Agreeableness (B = .11 99% CI [.06, .15], *dz* = .39, *ds* = .20), and Conscientiousness (B = .17 99% CI [.11, .23], *dz* = .48, *ds* = .28) remained significantly higher. In addition, Conscientiousness (B = − .07 99% CI [− .02, − .11], *dz* = − .22, *ds* = − .11) was significantly lower three months following ayahuasca ceremony compared to immediately following ayahuasca ceremony. With respect to informant-report data, main effects of timepoint were observed, such that, three months following ayahuasca ceremony, Neuroticism (F[1, 109] = 42.45, *p* < .0001, *mR*^2^ = .07, B = − .35 95% CI [− .45, − .24], *dz* = − .62, *ds* = − .39) was significantly lower, and Openness (F[1, 109] = 4.61, *p* = .03, *mR*^2^ = .01, B = .09 95% CI [.01, .17], *dz* = .20, *ds* = .13) was significantly higher. These results are displayed in Figs. 1 and 2, and facet-level results are displayed in Supplementary Figure 1.

### Examining moderation of personality change

To investigate the degree to which ayahuasca-induced personality change between timepoints depended on predisposing and experiential factors, linear mixed models were conducted in which moderators were separately added to the base model as fixed covariates. Five sets of moderator variables were examined including validity variables (including expectancies and MISS suggestibility), participant characteristics (including demographic variables, lifetime use of psychedelics), Baseline personality scores, acute experience elements, and ceremony variables (including ceremony characteristics, perceptions of ceremony, and purgative experiences). To reduce Type I error, a statistical significance threshold was set at *p* < .01 (*p* < .05 for hypotheses). Analyses focused on self-report personality. Unstandardized (B) coefficients indicate the added effect of the moderator to the effect of time. A summary of significant results from omnibus tests and change in marginal *R*^2^ after adding interaction terms is provided in Table 1. Full results are described in Supplementary Results I and Supplementary Table 1. Results that did not directly inform conclusions (e.g., moderation by participant characteristics) can be found in Supplementary Results II.

**Table 1:** Incremental variance explained by moderators.

Moderator	N	E	O	A	C	Moderator	N	E	O	A	C
Expectancies						Mystical Factor	**.026**	**.023**	.026	**.012**	**.011**
Increase in Spiritual	.007	.000	.002	.001	.003	RMEQ Mystical	**.029**	**.017**	**.037**	**.010**	**.015**
Increase in Connectedness	.002	.002	.003	.014	.013	RMEQ Positive Mood	**.033**	**.017**	**.034**	**.012**	**.013**
Decrease in N	**.118**	**.078**	.001	.001	**.027**	RMEQ Timespace	**.026**	.018	.038	.010	.029
Increase in E	**.027**	**.039**	.001	.000	**.018**	RMEQ Ineffable	**.004**	.004	.014	.004	.004
Increase in O	**.006**	.004	.002	.014	.020	Ego Dissolution Inventory	**.017**	**.007**	.009	**.011**	**.006**
Increase in A	**.011**	.004	.006	.001	.005	AEI Clarity	**.045**	**.035**	**.039**	**.008**	**.016**
Increase in C	.003	.002	.011	.002	**.025**	AEI Reappraisal	**.022**	**.015**	.042	**.004**	**.007**
Decrease in Anxiety	**.078**	.038	.004	.001	.002	AEI Discomfort	.024	**.015**	.010	.004	.005
Decrease in Depression	**.086**	**.054**	.006	.000	.020	Number of ceremonies	.005	.005	.007	.001	.007
Deal with inner conflict	.012	.001	.006	.000	.004	Average consumed	.004	.000	.006	.013	.006
Suggestibility	**.100**	.001	.007	.005	.059	Additional psychedelic	.002	.005	.003	.001	.005
Sex	.006	**.004**	.004	.073	.001	Retreat Length	.001	.001	.003	.001	.000
Age	**.004**	**.030**	**.035**	.016	**.010**	Trusted shaman	**.008**	.024	.058	.035	.004
Education Level	.013	.002	.001	.003	.023	Mesmerized by icaro prayer	.015	.014	.048	.089	.012
Parent Income	.008	.008	.005	.001	.009	Icaro prayer healing	**.011**	**.010**	.026	.066	.011
Psychedelic-naïve	.002	.004	.027	.001	.004	Medicine cleaning	**.014**	**.011**	.017	.010	**.005**
Ayahuasca-naïve	.006	.001	.003	**.008**	.002	Struggled to purge	.003	.001	.001	.010	.000
Baseline N	**.567**	**.224**	.002	.015	**.181**	War with entity	.017	.002	.002	.003	.008
Baseline E	**.184**	**.717**	.016	.003	.100	Purging self	**.028**	**.019**	.037	**.015**	**.021**
Baseline O	.003	.023	**.738**	**.065**	.002	Purged physical ailment	**.006**	.005	.003	.000	.002
Baseline A	.018	.003	.083	**.703**	.041	Purged completely	**.019**	**.009**	.010	.003	.002
Baseline C	**.160**	**.098**	.001	**.035**	**.646**	Viewed object	.004	.016	.001	.023	.003
						Relationship object	**.004**	.003	.005	.006	.010

*N* Neuroticism, *E* Extraversion, *O* Openness, *A* Agreeableness, *C* Conscientiousness, *RMEQ* Revised Mystical Experience Questionnaire, *AEI* Ayahuasca Experience Inventory.

Bolded marginal *R*^2^ values met *p* < .01 threshold.

**Figure 3:**
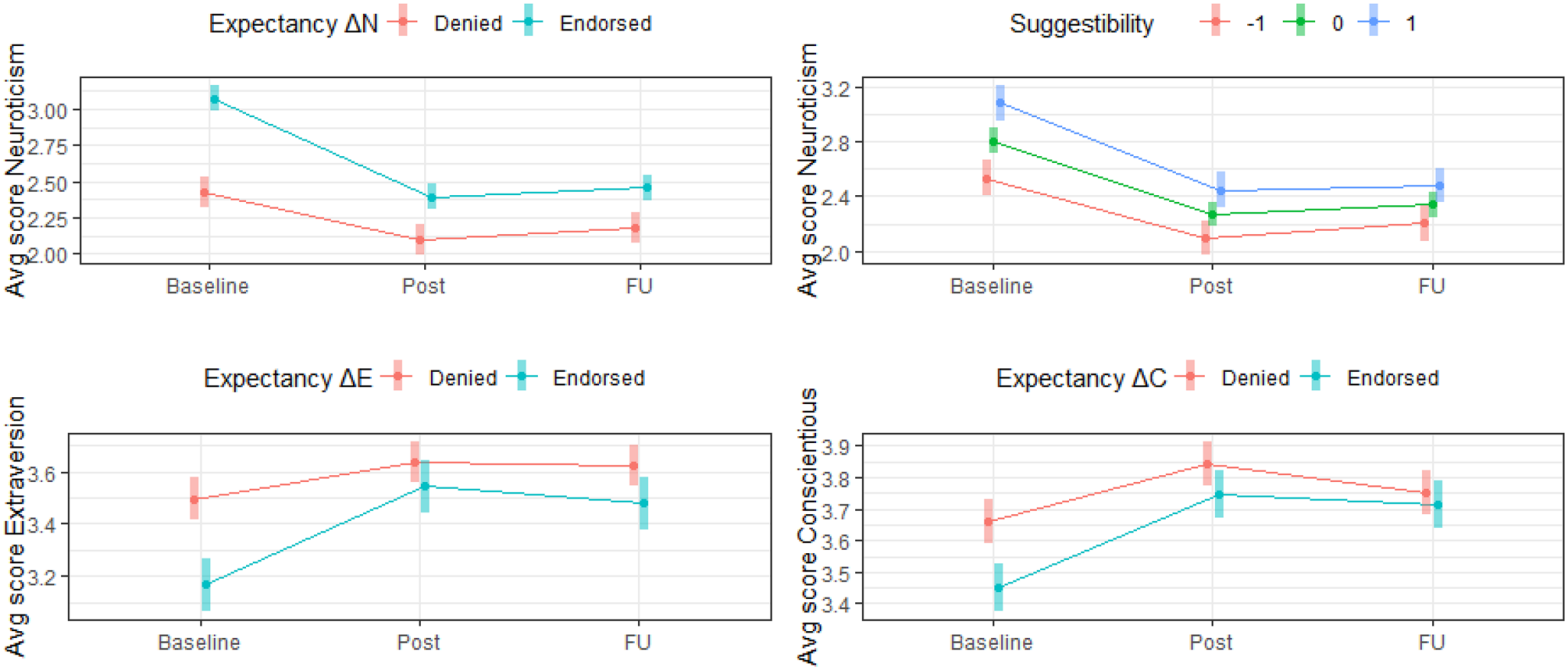
Line plots illustrating moderation of change in personality by validity variables. Top right figure illustrates change in Neuroticism at levels of Suggestibility corresponding to − 1, 0, and 1 standard deviations.

With respect to validity variables, two sets of variables were examined: expectancies of change and suggestibility. Expectancies involving favorable change in personality and psychopathology (e.g., anxiety, depression) showed evidence of amplifying change in Neuroticism, Extraversion, and Conscientiousness. Two notable patterns of change were observed. First, with respect to Neuroticism, participants endorsing an expectancy of favorable change in Neuroticism, depression, and anxiety exhibited higher baseline Neuroticism, and showed a greater decrease in Neuroticism following ceremony (B = − .35) and at Follow-up (B = − .37), compared to participants with lower expectancies. Similarly, participants endorsing an expectancy of favorable change in Extraversion and Conscientiousness exhibited lower baseline personality on respective domains, and showed a greater increase following ceremony (B = .24, .11, respectively) and at Follow-up (B = .18, .17, respectively). In addition, participants higher in suggestibility exhibited higher baseline Neuroticism, and showed a greater decrease in Neuroticism following ceremony and at Follow-up. Specifically, a one-standard-deviation increase in suggestibility was associated with an incremental .10 unit decrease in Neuroticism (on 5-point Likert scale) following ayahuasca ceremony and an incremental .14 unit decrease at Follow-up. These results are presented graphically in Fig. 3.

To examine whether change in personality was evident while controlling for validity variables, we examined self-reported change in personality among participants who endorsed no expectation of favorable change and who exhibited low trait suggestibility. Notably, statistically significant change in Neuroticism remained evident across self- and informant-report data among participants who denied expectancy (e.g., Expectancy_ΔN_: *dz*_selfT1–T3_ = − .59 [*N* = 106]; *dz*_informant_ = − .83 [*N* = 45], *p* < .01) and exhibited low suggestibility (indexed by scores below the 50th quantile; “somewhat disagree”; *dz*_selfT1–T3_ = − .67 [*N* = 138]; *dz*_informant_ = − .80 [*N* = 55], *p* < .01). Significant self-reported change in Extraversion was also observed among participants denying expectancy. However, a significant increase in Conscientiousness was not observed three months following ayahuasca ceremony for participants denying expectancy (B = .06, *t* = 2.24, *p* = .03), suggesting that expectancy effects may have influenced self-reported longer-term change.

**Figure 4:**
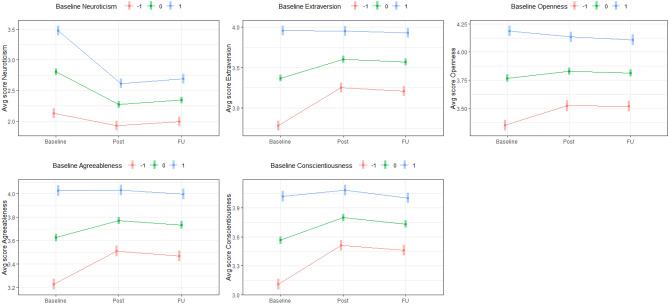
Line plots illustrating moderation of change in personality by baseline personality. Levels of baseline personality correspond to − 1, 0, and 1 standard deviations.

With respect to baseline personality, results indicated that all Baseline domain scores were significantly and substantially associated with adaptive changes in their respective personality domain. A one-standard-deviation increase in baseline personality significantly amplified change in Neuroticism, Extraversion, Openness, Agreeableness, and Conscientiousness by .33, .23, .13, .14, and .19 units (on 5-point Likert scale) three months following ayahuasca ceremony. Three notable patterns emerged in the data. First, participants showed a significant decrease in Neuroticism regardless of their standing on baseline Neuroticism. Second, participants higher (~ one-standard-deviation above others) in Extraversion, Agreeableness, and Conscientiousness showed no significant increase in these domains. Third, participants higher in Openness showed a significant decrease in Openness. These results are presented graphically in Fig. 4. Because the regression to the mean effect may provide a better explanation for these results, subsequent tests were conducted. Specifically, because extreme baseline scores are most vulnerable to regression, effect size estimates were examined while excluding participants above the 80th quantile of Baseline Neuroticism and below the 20th quantile of the other domains. Notably, the pattern of significant results for interaction effects and each level of the moderator remained the same for Neuroticism, Extraversion, Agreeableness, and Conscientiousness. However, for Openness, participants who reflected the mean of baseline Openness and above showed no significant increase in Openness over time.

**Figure 5:**
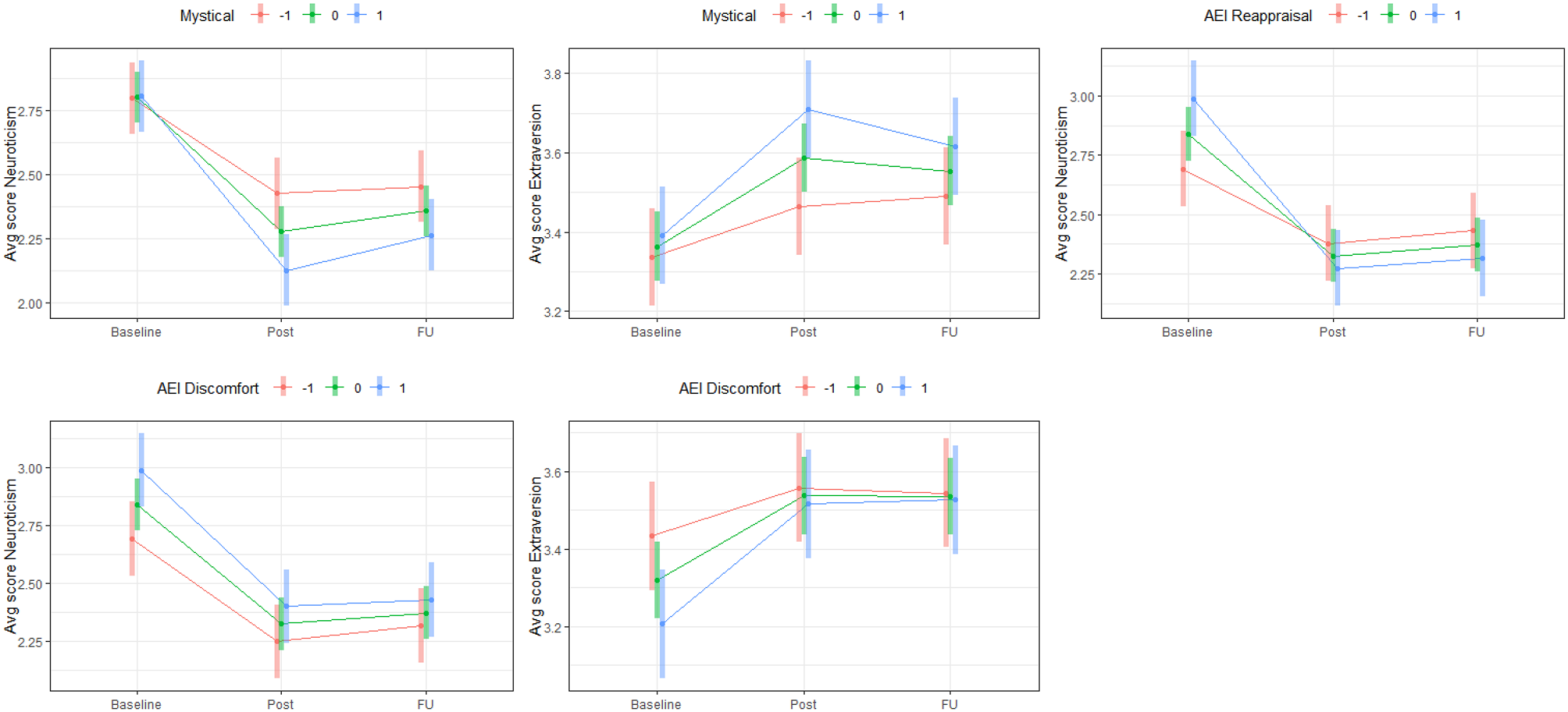
Line plots illustrating moderation of change in personality by experiential factors. Levels of experiential factors correspond to − 1, 0, and 1 standard deviations. *AEI* Ayahuasca Experience Inventory.

With respect to acute experience elements, we examined moderation at two levels of hierarchical structure. Factor analyses were conducted on (sub)scales of acute experience variables (e.g., RMEQ Mystical experience) to reduce redundancy (see Supplementary Tables 3, 4). AEI Discomfort emerged as one of two parsimonious factors, reflecting unpleasant feelings of torment, discomfort, and isolation; and a Mystical factor emerged (called Mystical hereafter), comprised of all other acute experience (sub)scales and broadly reflecting mystical-type experience and introspective reappraising of core self- and world-beliefs. With respect to Mystical, two notable patterns emerged. A one-standard-deviation increase in Mystical significantly (a) amplified decreases in Neuroticism following ayahuasca ceremony (B = − .15) and at Follow-up (B = − .10); and (b) amplified increases in Extraversion (B = .10), Openness (B = .04, *p* = .02), Agreeableness (B = .06), and Conscientiousness (B = .07) following ayahuasca ceremony (but not at Follow-up). Of note, the pattern of change associated with the Mystical factor for each domain was closely convergent with patterns associated with Mystical’s constituent variables. The one notable exception to this convergence involved AEI Reappraisal, which was the only variable to bear a significant association with initial levels of personality, namely Neuroticism (*r* = .22, *p* < .01), and showed the largest moderating effect on change in personality (B_T1–T3_ = − .21). Notably, despite beginning with higher Neuroticism, individuals who endorsed higher AEI Reappraisal experiences showed lower levels than their peers following ayahuasca ceremony and at Follow-up. With respect to AEI Discomfort, two notable patterns emerged. A one-standard-deviation increase in AEI Discomfort significantly (a) amplified increases in Extraversion following ayahuasca ceremony (B = .09) and at Follow-up (B = .10); and (b) amplified decreases in Neuroticism (B = − .09) at Follow-up. These results are presented graphically in Fig. 5.

**Figure 6:**
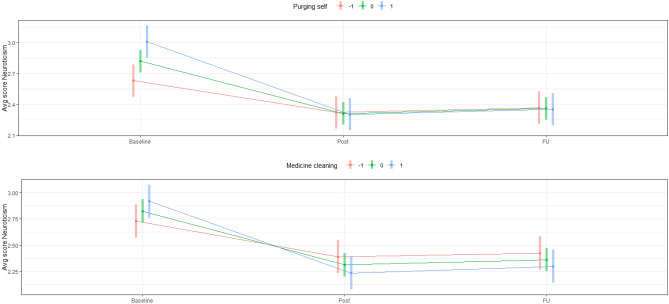
Line plots illustrating moderation of change in personality by ceremonial and purgative variables. Second plot is representative of moderation-based patterns of variables except Purging self. Levels of moderators correspond to − 1, 0, and 1 standard deviations.

Three sets of variables regarding the ceremonies were examined: (a) ceremony characteristics (e.g., Retreat length) (b) perceptions of ceremony (e.g., Trusted shaman), and (c) purgative experiences. No ceremony characteristics were significantly associated with change in FFM domains. With respect to perceptions of ceremony and purgative experience variables, two general patterns were notable: First, participants endorsing experiences involving purging a negative part of themselves were (a) more likely to be higher in Neuroticism and lower in Extraversion and Conscientiousness at Baseline; and (b) tended to exhibit similar levels of these traits to other participants following ayahuasca ceremony and at Follow-up. Second, participants endorsing higher levels of the other significant variables (e.g., regarding ayahuasca decoction as medicinal, regarding one’s purgative experience to be complete and satisfying, see Table 1) tended to show similar initial levels of personality and exhibit greater changes than other participants. These results are presented graphically in Fig. 6.

This pattern of moderation-based results was generally not supported across equivalent analyses in the informant-report sample, though suboptimal statistical power limits confidence in these results (see “Methods” section).

## Discussion

The present study prospectively explored the ceremonial use of ayahuasca as a potential driver of personality change. Two main questions were examined: (a) Is ceremonial use of ayahuasca related to changes in self- and informant-reported personality; and (b) Are there factors that predispose or potentiate change in personality? Given our inability to implement a control condition with placebo, we employed a series of methodological safeguards to reduce placebo, expectancy, and demand threats. First, participants were excluded who showed a higher risk of inaccurately endorsing personality change and/or over-endorsing experiential phenomena in ceremony. Second, in view of significant placebo and expectancy effects on clinical outcomes^58,59^ and demonstrated placebo effects on the presence of psychedelic states^60^, expectancies of favorable change and trait suggestibility were measured and examined in our analyses. Third, because placebo effects are likely to decline following retreat experience, personality was measured immediately after use as well as three months following retreat. Finally, to partially circumvent placebo, expectancy, and demand effects, and provide resolution on traits that may be more accurately described by observers^61^, corroborating informant-report data were measured.

### Does ceremonial use of ayahuasca prompt change in personality?

Neuroticism was observed to decline substantially (.62 standard deviations in baseline neuroticism scores [*ds*]; 1.00 standard deviations in change scores [*dz*]) between pre-retreat measurement and the week following ayahuasca ceremony, and remain substantially below pre-retreat scores (decline of .53 standard deviations in neuroticism scores [*ds*]; .85 standard deviations in change scores [*dz*]) three months following ceremony. Notably, the decline was also reflected in informant-report data. These results are consistent with a number of previous prospective findings including effects of ayahuasca on worry, self-directedness, major depression, and neuroticism^35,37,38,43,44^, and effects of psilocybin-assisted therapy on major depression and neuroticism^1,3,46^. The results also converge with multiple cross-sectional findings demonstrating lower harm avoidance and neuroticism among long-term practitioners of ayahuasca ceremony versus controls^62–65^.

Interpreting the magnitude of the effect on neuroticism may be best supported through comparison to meta-analyzed effects of clinical interventions. In a large meta-analysis (*k* = 199; N =  ~ 20,000), Roberts and colleagues^28^ estimated a .57 average pre-post effect size decline in self-report neuroticism following intervention (compared to .79 in the present sample when calculated in an equivalent manner). Pertinent to the present examination, the authors observed that interventions lasting less than four weeks tended to have small effects. By comparison, self-reported change in Neuroticism following an average of 1.4 weeks at the retreat center and 4.4 ayahuasca ceremonies was associated with an effect comparable to the average effect of multiple weeks of clinical intervention. The present results are also consistent with previous findings for psychedelic-assisted therapy including Erritzoe and colleagues’^1^ observation of a medium-sized reduction in neuroticism at 3 months follow-up (*d* = − .57; compared to − .53 in our sample when calculated in equivalent manner).

Support for hypotheses predicting change in extraversion and openness was not found as robustly in that although domain openness notably exhibited statistically significant change within self- and informant-data, significant differences between timepoints were not consistently observed while also accounting for expectancy effects and suggestibility. We acknowledge, nevertheless, that restricted sample size within these tests may have artificially contributed to null results. A ceiling effect may also have limited upward change in openness as we observed clearly elevated levels of mean baseline openness in the present sample (mean = 3.78) relative to normative samples (see Supplementary Table 6). In other words, ceremonial ayahuasca activity in these settings may be particularly likely to select for high openness individuals.

### Are there factors that predispose individuals to change in personality?

As psychedelic-assisted therapies gain greater acceptance as practical tools for clinical treatment, one question is whether individual difference factors predispose positive or negative outcomes. Our major finding was that baseline personality emerged as a strong moderator of adaptive changes in personality across domains. Specifically, higher levels of neuroticism and lower levels of extraversion, openness, agreeableness, and conscientiousness seemed to predispose participants to larger adaptive changes—that is, those with less psychologically adaptive trait scores^97^ demonstrated the most change towards greater adaptivity in trait scores. The regression to the mean effect looms as a possible explanation for this finding. However, our attempts to eliminate extreme scores most susceptible to mean regression did not substantively weaken personality change effects. There are a number of implications of this tentative finding. Personality change effects may strongly depend on an individual’s initial standing on personality. Because the results of research studies would similarly depend on the mean level and variance of participant personality contained in their samples, baseline personality may substantively account for variability in extant personality change-related findings, and notably, explain why Erritzoe and colleagues’^1^ sample containing individuals with treatment-resistant depression showed change in neuroticism whereas other studies have not.

### Are there experiential factors during ayahuasca ceremony that affect change in personality?

Unlike most pharmacological interventions, psychedelic administration accompanies non-ordinary states of consciousness whose quality and intensity may influence subsequent outcomes. Perhaps our most important finding is that mystical-type experiences were associated with larger adaptive changes in all personality domains. These results were partially consistent with previous literature demonstrating moderating effects on neuroticism, extraversion, and openness^1,2^. Factor loadings from the Mystical factor were suggestive that sensing unification with a larger whole, reverence, intuitive insight, peace, self-connection/acceptance/love, centeredness, and trust may broadly capture the core of ayahuasca-induced mystical experience. Of note, something like this core has been described across multiple spiritual and psychological traditions (e.g., Hinduism’s Atman [true-self]; Stace’s^66^ components of spiritual experience; Freud’s^67^ “oceanic feeling” and “bond with the universe;” Jung’s^68^ “revelatory” states in the unconscious; Maslow’s^69^ peak-experience).

Second, AEI Reappraisal, a construct reflecting introspective reappraising of difficult life experiences and negative core beliefs during ayahuasca ceremony, emerged as the strongest experiential moderator of change in neuroticism (B_T1–T3_ = − .21). This construct is notable in resembling core elements of psychotherapy such as testing the accuracy of existing beliefs^70^, deriving new meaning from past trauma^71^, confronting fears and enacting courage^70^, meta-cognition on otherwise unconscious patterns of thinking, feeling, and behaving^72^, psychological flexibility, and orienting toward values, meaning, and growth^73–75^. It will be important for future research to examine the degree to which mechanisms of change within psychedelic experience naturally facilitate adaptive processes that converge with those of Western psychotherapy.

Third, support for the influence of certain ceremonial and purgative elements (e.g., trusting the shaman) raises questions about the unique value of a shamanic context for personality change. Possible explanations for the effects of shamanic elements include (a) the activity of medicinal/healing elements of a psychospiritual nature; (b) a heightened mystical state of consciousness that covaries with favorable attitudes toward and perceptions of shamanic elements; and/or (c) a higher susceptibility to placebo. Secondary analyses provided strongest support for (b) and negligible support for (c). However, it bears noting that the Medicine cleaning and Purging part of self items remained significant predictors (*p* < .01) of longer-term change in neuroticism and extraversion while controlling for both sets of covariates. It has long been queried whether the purgative elements unique to ayahuasca ceremony produce additive therapeutic effect. This result may be suggestive that some purgative elements are additive. However, our results were generally suggestive that this was not the case for most measured shamanic elements.

### What can be learned from ayahuasca ceremony?

The present study shows preliminary support for the therapeutic benefit of the shaman, icaro, purgative elements, cognitive reappraisal, sacramental atmosphere, and communal/group context. Parallels between these elements and modern psychedelic-assisted protocols are notable. In therapeutic protocols from Johns Hopkins and Imperial College London, carefully curated music is selected to guide research participants^76^, and therapists, like shamans, unobtrusively guide the participant while encouraging inward focus^77^. Even so, ayahuasca ceremony may contain additional therapeutic components. First, the live, visceral presence of ceremonial music, and its convergence with the guide’s (i.e., shaman, therapist) other roles may produce stronger mystical-type experiences. Second, a communal, group-based format^78^ has potential benefits of normalizing psychological struggles; promoting empathy, bonding, and trust-building as individuals vulnerably confront adversity; and affording constructive opportunities to enact new behavioral patterns of interpersonal relating.

The present results also provided tentative support for the role of shamanic and purgative elements in shaping adaptive outcomes. There could be elements of shamanic philosophy (e.g., connection with the natural world) that are psychologically beneficial, and it should not be ruled out that psychospiritual agents, understood within the shamanic metaphysics, guide adaptive changes. Whether elements of ayahuasca ceremony hold additive benefit over and above Western components deserves ongoing attention^75^.

## Limitations

Notable limitations include the absence of a placebo-control group or blinding protocols. The naturalistic approach of the present study precluded the use of a control group, raising the potential for significant methodological issues including the influence of placebo, expectancy, demand, and Hawthorne effects. It is possible that merely attending a therapeutically designed retreat in a foreign country would in and of itself produce positive changes to personality. Second, given the uniqueness of the present sample (e.g., high baseline openness), the generalizability of these findings requires careful consideration, and replication in samples with different characteristics are needed. Third, a portion of participants who were unresponsive to survey invitations were incentivized with additional monetary compensation to lower attrition. This approach may have biased their responses. Nonetheless, the number of such participants was low (N = 6, 2% of sample), and these participants did not differ from others in baseline personality scores (*p* < .05). Fourth, it will be important for future research to investigate the duration of these positive personality states over longer periods of time given that many contemporary antidepressant options show diminishing effects over time^79^.

## Conclusion

The present study represented a relatively well-powered examination of personality change in relation to ayahuasca ceremony. Attending ayahuasca ceremony was associated with a medium-sized decrease in neuroticism, and our results pointed to predisposing and experiential factors that may potentiate personality change. Attributes of ayahuasca ceremony may be particularly powerful as a treatment for neuroticism/internalizing psychopathology, especially among individuals at elevated baseline levels. Furthermore, baseline personality, mystical-type experiences, meta-cognitive reappraisal, and certain shamanic elements emerged as predictors of favorable personality change. Implications of the present study include the importance of (a) well-powered samples and tests of moderation; (b) using samples lower in extraversion, openness, agreeableness, and conscientiousness and higher in neuroticism; (c) using safeguards against Type I error including larger samples, informant-report data, and validity controls; and (d) continuing to investigate the degree to which psychedelic-induced changes in negative core beliefs underlie changes in neuroticism.

## Methods

### Participants and procedure

Three-hundred-thirty participants were recruited from three ayahuasca retreat centers across South and Central America: Arkana Spiritual Center (Requena, Loreto, Peru), Soltara Healing Center (Gulf of Nicoya, Costa Rica), and La Medicina (Cordilliera Escalera mountain range, Peru). Compensation involved entry into a raffle for a week-long retreat at Arkana Spiritual Center (valued at $1580.00) and further incentives were used to promote compliance and reduce attrition. Of the 330 participants who were recruited, 3% (*N* = 10) met criteria for invalid responding based on the Elemental Psychopathy Assessment validity scales and inadequate time-commitment (i.e., < 25% of average time contributed to personality questionnaire), leaving a sample of 320 validly responding participants possessing data at at least one timepoint. Data were furthermore removed in pair-wise fashion for seven participants on the basis of inadequate time-commitment and untimely completion. The final sample consisted of 256 participants (80% of valid respondents) who provided data for all three timepoints (161 males and 94 females; mean age = 34.8 [SD = 9.9]; 81% White, 2% Black, 5% Asian, and 7% Hispanic, 2% Native American, 2% Other). All participants provided informed consent in accordance with the Common Rule and the Declaration of Helsinki. All procedures were approved by the University of Georgia Institutional Review Board.

Independent samples t-test analyses were conducted to test for differences between validly responding participants who missed one or more timepoints (*N* = 67), and validly responding participants with no missing data (*N* = 252). At Baseline, participants with missing data had lower Conscientiousness scores (*ds* = .34, *p* < .05).

Of validly responding participants, 33% (*N* = 104) possessed informant-report measurement at Baseline and Follow-up, and an additional six cases of informant data from invalidly responding participants supplemented this sample, yielding an informant-report sample of 110 participants. Data was only included from informants who reported at both Baseline and Follow-up timepoints to reduce error due to imperfect consensus between informants. Data was computed by averaging across informants at the item-level for each timepoint. The number of informants per participant ranged from one to three. Independent-samples t-test analyses were conducted to test for differences between final sample participants with informant data (*N* = 104) and final sample participants without informant data (*N* = 165). No significant differences were found.

#### Retreat center experience

Arkana Spiritual Center, Soltara Healing Center, and La Medicina host shamans from the Shipibo lineage (originating geographically in the west Amazon basin). Among the ceremonies offered to retreat clients, the ayahuasca ceremony (offered approximately four times each week) represents the most time-intensive and immersive practice. Ayahuasca ceremony and communal check-ins the following day are conjectured to contribute most meaningfully to the observed effects. The ceremonial use of nunu (tobacco and ash snuff), flower baths, kambo (a purgative frog venom), sapo (Bufo Alvarius toad venom containing 5-methoxy-N,N-dimethyltryptamine) and huachuma (or San Pedro cactus containing 3,4,5-trimethoxyphenethylamine mescaline), among others, are also offered. Study procedure is provided in Supplementary Methods I.

## Measures

### Outcome measure

#### Five-factor model personality

A 120-item set of the International Personality Item Pool (IPIP-NEO-120^80^) was used to index self-reported personality traits. The IPIP-NEO-120 consists of five 24-item FFM domain subscales, and 30 4-item FFM facet subscales, and has demonstrated good reliability and construct validity when compared to the Revised NEO Personality Inventory^80,81^. A 60-item set of the International Personality Item Pool (IPIP-NEO-60^78^) was used to index informant-reported traits. The IPIP-NEO-60 consists of five 12-item FFM domain subscales, and 30 2-item FFM facet subscales, and has demonstrated good reliability and construct validity^82^. Of note, self- and informant-report data are regarded to show adequate measurement invariance for comparing relative standings of individuals^83^. FFM domains have shown adequate test–retest reliability across an average interval of four weeks (*r*s > .77)^84^. Longitudinal measurement invariance has also been supported at the metric and scalar level in large samples^85,86^. Internal consistency ranged from .79 (Openness Follow-up) to .93 (Neuroticism Baseline) for self-report data, and from .71 (Openness Follow-up) to .87 (Extraversion Baseline) for informant-report data. Facet-level internal consistency is provided in Supplementary Table 2.

### Evaluation of validity

#### Suggestibility

The Multidisciplinary Iowa Suggestibility Scale-Short (MISS^87^) was used to measure participants’ susceptibility to internalize external influences. The MISS is a 21-item self-report scale that uses a 5-point likert scale.

#### Expectancies

An original scale was developed, consisting of ten dichotomous items measuring expected change in personality domains (e.g., “I will become more open to experience, i.e., will become more intellectually curious, open to emotion, sensitive to beauty and/or willing to try new things”), spirituality (“I will become more spiritual”), and internalizing symptoms (e.g., “I will experience a significant reduction of depressive thinking”).

#### Experiential factor validity items

An original 3-item scale was used to measure overreporting of acute mystical-type experiences. Participants were asked the degree to which they experienced the following low base-rate phenomena: “Experience of a distant childhood friend you have not seen or thought of in a long time,” “Rapidly fluctuating pattern of feelings alternating from joy to sadness and back again,” “Experience of bodily fragmentation, such that parts of your body are separated from one another.” The cut-off for invalid responding was defined as strong or extreme endorsement of all three validity items. On the basis of this cut-off, data for measures indexing acute experience was excluded for 11 participants.

#### Invalid responding

Two 8-item validity scales from the Elemental Psychopathy Assessment^88^ were used to detect invalid responding on measures of personality. These scales were the Infrequency scale and the Unlikely Virtue scale. In line with guidelines^88^, participants who endorsed more than three Infrequency scale items and more than two Unlikely Virtue scale items were eliminated.

#### Participant characteristics

Participant characteristics including sex, age, personal education level, and parents’ income level were measured. Education level was a 7-point ordinal variable ranging from “Less than 7 years of school” to “Doctoral Degree.” Income level was a 14-point ordinal variable ranging from “$0–$5000” to “$120,000 or more” in increments of $10,000.

#### Lifetime use of psychedelic compounds

Participants were asked to report previous use of classic psychedelic compounds and previous ceremonial use of ayahuasca. These variables were dichotomized to reflect the presence or absence of previous psychedelic experience. Complete data for lifetime use of ayahuasca was available for 230 participants.

### Experiential factors

#### Mystical experience

The Revised-Mystical Experience Questionnaire (RMEQ^56,89^) was used to assess mystical aspects of participants’ experiences during ayahuasca ceremony. The RMEQ consists of thirty items originally represented on the Pahnke-Richards Mystical Experience Questionnaire^53,90^. In line with psychometric work^89^, four subscales were assessed: Mystical (15-item; e.g., “Experience of the fusion of your personal self into a larger whole”), Positive mood (6-item; e.g., “Sense of awe or awesomeness”), Transcendence of time and space (6-item; e.g., “Loss of your usual sense of space”), and Ineffability (3-item; e.g., “Sense that the experience cannot be described adequately in words”). Items asked participants to consider the degree to which they had experienced the preceding phenomena at any time during the ceremony. Items used a 6-point Likert scale. One of the items from the Mystical subscale (RMEQ item 30) was excluded due to administrator error. Internal consistency (α) ranged from .88 (Ineffability) to .96 (Mystical). Complete data were available for 237 participants.

#### Ego dissolution

The Ego Dissolution Inventory (EDI^91^) was used to measure dissolution of ego during the acute effects of ayahuasca. The EDI consists of eight items (e.g., “I experienced a disintegration of my ‘self’ or ego”) using a 5-point Likert scale to measure the presence of dissolution phenomena. Internal consistency was good (α = .89). Complete data were available for 237 participants.

#### Ayahuasca Experience Inventory

Qualitative methods are regarded as highly informative when observing new experiential phenomena because they “allow for identification of previously unknown processes, explanations of why and how phenomena occur, and the range of their effects”^92^. Using these methods, the Ayahuasca Experience Inventory (AEI^93^) was developed to measure thoughts, feelings, behaviors, and attitudes that arise within ayahuasca ceremonies. The AEI consists of three factors: Clarity (32 items) captures clarity, peace, self-connection, and self-esteem; Reappraisal (30 items) captures cognitive reappraisal of negative beliefs about self/others, and initiative to enact life changes; and Discomfort (15 items) captures unpleasant feelings of torment, discomfort, and isolation that seemed unending. Items asked participants to consider the degree to which they had experienced phenomena at any time during ceremony. Items were measured using a 6-point Likert scale. Participant scores, using Thurstone regression-based weighting, were generated from factor analyses on the same data as used herein. Model fit results, loadings, and items are provided in Supplementary Tables 9 and 10. AEI factors exhibited internal consistency (α) ranging from .94 (Discomfort) to .97 (Clarity). Complete data were available for 188 participants.

#### Shamanic elements

Shamanic element items (11 items)^93^ were generated using the same method as the AEI, and were administered to capture participants’ perceptions of the shaman, icaro prayer, and ayahuasca brew, and experiences related to purging. Response options mirrored the AEI and RMEQ. Items are provided in Supplementary Table 11. Complete data was available for 195 participants.

#### Ceremony characteristics

Participants were asked about characteristics of their retreat experience including the number of ceremonies in which they consumed ayahuasca, retreat length, average dosage of ayahuasca, and experiences with other plant medicines (e.g., huachuma, sapo). Dosage was computed as the approximate ayahuasca quantity consumed across ceremonies in terms of glasses (e.g., one and one-half glasses). Of note, glass size was not standardized and varied within and across retreat centers. Complete data for dosage and frequency of ceremonies were available for 242 participants. Complete data concerning other plant medicines used were available for 222 participants.

#### Reducing experiential variables

To reduce redundancy among related experiential variables, exploratory factor analyses were conducted on experiential factor subscales (RMEQ subscales, EDI, AEI subscales; Supplementary Methods II). The first factor reflected all subscales besides AEI Discomfort, thus capturing mystical experience and reappraisal (referred to as Mystical); the second factor consisted of AEI Discomfort. Complete data was available for 237 participants.

### Analytic plan

#### Preregistration

Hypotheses and analyses were preregistered using the Open Science Foundation web platform (https://osf.io/xk3ym). However, two deviations from our original plan are notable (Supplementary Methods III).

#### Analyses

Five sets of analyses were planned. The first set of analyses examined the degree to which personality changed in relation to ayahuasca ceremony. Linear mixed models were conducted (equivalent to one-way repeated measures ANOVA) to determine the persisting effects of ayahuasca ceremony on self- and informant-report personality measures, comparing each measure between each timepoint (Baseline, Post, and 3-month Follow-up). Where a significant main effect was observed, post-hoc comparisons were conducted between each timepoint without corrections for multiple comparisons. Cohen’s *ds* (standard *Cohen’s d*)^94,95^ effect size estimates were calculated using the following equation: (Mean-score_T2_–Mean-score_T1_)/((SD_T1_)^2^ + SD_T2_)^2^)^0.5^. Cohen’s *dz*^95^ effect size estimates (for one-sample within-subjects designs) were calculated by dividing the mean difference of personality scores between two timepoints by the standard deviation of differences in scores between the same two timepoints. The second set of analyses used linear mixed models to examine moderating effects on time. Linear mixed effects models were conducted in which we included moderators as fixed covariates to the base model (including time). The third set of analyses examined relations between self- and informant-reported FFM domains to confirm convergence between the two approaches and validate informant-reported scores (Supplementary Results III). The fourth set of analyses examined the distributional properties (mean, variance) of Baseline FFM personality scores in the present sample, and compared these properties to normative populations to delineate the uniqueness of the sampled population (Supplementary Results IV). The fifth set of analyses examined zero-order correlations among Baseline personality domains and experiential factors (Supplementary Results V).

#### Power analyses

Post-hoc power analyses (using ‘simr’ package in R) were conducted to assess power for the first and second sets of analyses. For each set of analyses, effect sizes sufficient to obtain 80% statistical power (alpha value *p* = .01, using 100 Monte Carlo simulations) were estimated. Results indicated that the self-report sample was powered (80%) to accurately detect true differences between timepoints exceeding .11 (Neuroticism), .15 (Extraversion), .15 (Openness), .15 (Agreeableness), and .16 (Conscientiousness) standard deviations; and the informant-sample was powered (80%) to accurately detect true differences between timepoints exceeding .30 (Neuroticism), .26 (Extraversion), .28 (Openness), .27 (Agreeableness), and .26 (Conscientiousness) standard deviations. With respect to moderation-based analyses, the self-report sample was powered (80%) to detect true interaction effects of small size (standardized interaction coefficient effect size ranged from .14 to .23 across domains and moderators); and the informant-report sample was powered (80%) to detect true interaction effects of small to medium size (standardized coefficient effect size ranged from .25 to .33 across domains and moderators).

## Supplementary Information

Supplementary Informations.
