# Supplementary material for: Examining changes in personality following shamanic ceremonial use of ayahuasca
Source: Sci Rep. 2021 Mar 23;11:6653. doi: 10.1038/s41598-021-84746-0 (PMC7987971; doi:10.1038/s41598-021-84746-0)
Supplement: Supplementary file 1 — Supplementary Informations. [file 41598_2021_84746_MOESM1_ESM.docx]

Running Head: AYAHUASCA PERSONALITY CHANGE SUPPLMENTARY

**Examining changes in personality following shamanic ceremonial use of ayahuasca**

Brandon Weiss*

Joshua D. Miller

Nathan T. Carter

W. Keith Campbell

University of Georgia

Corresponding author:

Brandon Weiss (bw64357@gmail.com)

**Supplementary Materials**

**Supplementary Methods I: Study Procedure**

Participants were emailed two weeks before the date of their ayahuasca retreat with an invitation to enroll. Participants were informed that they would be compensated with a customized personality change report and entry into a raffle for a week-long retreat at Arkana Spiritual Center. The Baseline survey asked participants to include contact information of close significant others (informants), who were subsequently contacted with an informant survey. On average, participants filled out the Baseline survey eight days before the beginning of the retreat (SD=6.85 days).

On the first day of retreat, participants were provided with a link to the Post survey. On the last day of retreat, participants were reminded to complete the Post survey. Of two reminder emails, the second email offered participants $20.00 compensation for completing the survey. On average, participants filled out the Post survey five days following retreat end (SD=4.93 days).

Three months following the last day of retreat, participants were provided with the Follow-up survey, and informants were invited to rate target participants’ personality for the second time. Up to three reminder emails were subsequently sent. The second and third reminder emails offered an incentive of $20.00 and $30.00, respectively, to complete the survey. On average, participants filled out the Follow-up survey 18 days following their invitation (SD=18.32 days).

**Supplementary Methods II: Reducing Experiential Variables**

To reduce redundancy among related experiential variables and derive parsimonious psychological factors of acute psychological experience, exploratory factor analyses were conducted on experiential factor subscales (RMEQ subscales, EDI, AEI subscales) using a principal axis factoring method with promax rotation. A parallel analysis^1^ showed that only two factors explained meaningful variance beyond simulated factors. As such, models containing up to two factors were considered. Although Velicer’s Minimum average partial (MAP)^2^ achieved a minimum of .07 with one factor, Very Simple Structure (VSS) indicated that two factors optimized model fit. The two-factor model was selected due to favorable SRMR, TLI, and BIC values, meaningful conceptual differences between the two factors (i.e., AEI Discomfort showed a negligible loading onto the first factor), and meaningful variance explained by the second factor (i.e., 20%). Model fit statistics are provided in Supplementary Table 3, and loadings are provided in Supplementary Table 4. The first factor reflected all subscales besides AEI Discomfort, and thus captured mystical experience and reappraisal; the second factor consisted of AEI Discomfort.

**Supplementary Methods III: Preregistration note**

Hypotheses and analyses were preregistered using the Open Science Foundation web platform (https://osf.io/xk3ym). Two deviations from our original preregistered plan are notable. First, the plan was to examine fine-grained personality aspects^3^ in order to detect differential functioning of meaningfully distinct aspects within each domain while avoiding redundancy in facet-level analysis. However, hierarchical confirmatory factor analytic (CFA) models in which IPIP-120 items relating to each FFM domain were loaded onto FFM facets, and FFM facets were loaded onto two related aspects (in line with meta-analytic work by Judge et al.^4^) resulted in poor model fit (according to Hu and Bentler’s^5^ close-fit standard) across the majority of FFM domains. Exploratory factor analytic (EFA) models similarly provided weak support for two-factor models in line with the aspect structure. As such, the six-facet structure of the original IPIP-120 was used for analyses. Second, our original plan was to examine change in personality and moderation of personality change using a structural equation modeling (SEM) framework. However, preliminary evaluations of model fit for latent growth curve models in which Baseline, Post, and Follow-up personality scores were regressed on linear Time yielded very poor fit across FFM domains. Reasons for poor fit could reasonably include (a) uneven increments of time between Baseline, Post, and Follow-up measurement points; (b) a quadratic course of change (given the likelihood of lower incremental change between Post and Follow-up) that could not be examined using only three measurement points; and/or (c) small sample size. Accordingly, we examined only two measurement points at a time in paired-sample t-test and regression analyses.

**Supplementary Results I: Results of Linear Mixed Models**

Results are presented for three sets of analyses (Supplementary Table 1). The first set of analyses (Main effect of Time [self-report]) used linear mixed models to examine specific differences in self-reported personality between Baseline, Post, and Follow-up. Coefficients indicate average personality at Baseline (Intercept), and estimated differences in personality between Baseline and Post (Time Post) and between Baseline and Follow-up (Time FU). The second set of analyses (Main effect of Time [informant-report]) used linear mixed models to examine differences in informant-reported personality between Baseline and Follow-up. Coefficients indicate average personality at Baseline (Intercept), and the estimated difference in personality between Baseline and Follow-up (Time FU). The third set of analyses (Moderation [self-report]) used linear mixed models to examine the degree to which moderator variables augmented specific effects of Time on self-reported personality between timepoints. Only moderators that showed statistically significant effects in omnibus ANOVA tests (see Table 1 in main text) were probed. For continuous moderators, coefficients indicate average personality for participants exhibiting mean levels of the moderator at Baseline (Intercept), the linear association between the moderator and personality (Moderator), the estimated difference in personality between Baseline and Post controlling for the effect of the moderator (Time Post), the estimated difference in personality between Baseline and Follow-up controlling for the effect of the moderator (Time FU), the estimated degree to which a one standard deviation increase in the moderator affects the Time Post effect (Moderator X Time Post), and the estimated degree to which a one standard deviation increase in the moderator affects the Time FU effect (Moderator X Time FU). For categorical moderators, coefficients indicate average personality for participants within the first (of two) level of the moderator at Baseline (Intercept), the linear association between the moderator and personality (Moderator), the estimated difference in personality between Baseline and Post controlling for the effect of the moderator (Time Post), the estimated difference in personality between Baseline and Follow-up controlling for the effect of the moderator (Time FU), the estimated degree to which a one unit increase in the moderator affects the Time Post effect (Moderator X Time Post), and the estimated degree to which a one unit increase in the moderator affects the Time FU effect (Moderator X Time FU).

**Supplementary Results II: Moderation by participant characteristics**

With respect to participant characteristics, two sets of variables were examined: demographic and lifetime substance use. Sex, age, and lifetime use of ayahuasca emerged as significant moderators. Male participants exhibited a greater increase in Extraversion following ayahuasca ceremony (B=.16, M_diffmalesT1-T2_=.46, M_difffemalesT1-T2_=.13). Younger participants exhibited greater change in Neuroticism (lower), Extraversion (higher), Openness (higher), and Conscientiousness (higher) following ayahuasca ceremony, but not three months following ayahuasca ceremony. Lastly, ayahuasca-naïve participants exhibited a greater increase in Agreeableness following ayahuasca ceremony than participants with previous experience (B=.13, M_diffaya-naive_=.29, M_diffaya-exp_=.03).

**Supplementary Results III: Self- and Informant-report correlations**

Scores on self- and informant-reported FFM domains were correlated to determine convergence between self- and informant-report data at Baseline and Follow-up. Results are provided in Supplementary Table 5. Results indicated moderate to strong convergence between corresponding domains and measurement points. Extraversion showed substantial convergence for both measurement points (*r*_T1_ = .66; *r*_T3_ = .61) in line with work demonstrating enhanced informant-report accuracy for observable traits^6^. Notably, Neuroticism showed better than expected convergence across measurement points (*r*_T1_ = .52; *r*_T3_ = .41) relative to meta-analytic estimates (i.e., *r* = .32)^7^. Openness, Agreeableness, and Conscientiousness generally showed moderate self-other convergence (Openness: *r*_T1_ = .53; *r*_T3_ = .33; Agreeableness: *r*_T1_ = .38; *r*_T3_ = .31; Conscientiousness: *r*_T1_ = .55; *r*_T3_ = .35).

**Supplementary Results IV: Sample properties**

To understand the distributional properties of the present sample, means and variance for Baseline FFM personality domains and facets were compared to the properties of two normative samples: an undergraduate sample (*N* = 359)^8^ and a community sample (*N* = 501)^9^. Values are contained in Supplementary Tables 6 and 7. Reported differences were statistically significant at *p* < .005. With respect to mean properties, Baseline Neuroticism scores were more elevated than in the undergraduate sample (*d* = .28) and the community sample (*d* = .73); Baseline Extraversion scores were lower than the undergraduate sample (*d* = .72), but not the community sample; Baseline Openness scores were substantially more elevated than in the undergraduate sample (*d* = .85) and community sample (*d* = 1.02); Baseline Agreeableness scores were lower than in the undergraduate sample (*d* = .15) and community sample (*d* = .66); and Baseline Conscientiousness scores were lower than in the community sample (*d* = .91), but not the undergraduate sample. In sum, the sampled population of ayahuasca retreat-goers exhibited meaningfully elevated Neuroticism and Openness, and lower Agreeableness and Conscientiousness than the general population. These observations should however be qualified by the suboptimally small size of our normative samples.

With respect to variance properties, the standard deviations of FFM domains were generally comparable to normative samples with the exception of Baseline Neuroticism and Openness which exhibited higher and lower standard deviations, respectively (diff_SD_ > .10). Collectively, observations regarding mean and variance properties of Baseline FFM data were suggestive of broader variability in and higher mean Neuroticism scores and more restricted variability and higher mean Openness scores than the general population. Whereas the distribution of Neuroticism scores was more conducive to uncovering personality change trends if they existed, the distribution of Openness scores was suggestive of possible restriction of range issues with respect to elevations in already high scores over time.

**Supplementary Results V: Relations among Baseline Personality and Experiential Factors**

To investigate relations between Baseline FFM domains and experiential factors, correlations among variables were calculated using a statistical significance threshold of *p* < .005 to control for Type I error^10^. Results are provided in Supplementary Table 8. In describing effect size, small, moderate, and strong refer to Cohen’s^11^ benchmarks (i.e., *r* = ~.10, ~.30, ~.50, respectively). First, the pattern of intercorrelations among FFM domains was consistent with meta-analytic findings,^12^ with some notable deviations. Correlations between Neuroticism, on one hand, and Extraversion and Conscientiousness, on the other, were stronger in magnitude (*r* = -.58, -.51, respectively, [versus -.26, -.32 in meta-analysis]); the correlation between Extraversion and Conscientiousness was larger in magnitude (*r* = .37 [versus .21]); and the correlation between Openness and Agreeableness was larger in magnitude (*r* = .31 [versus .14]). Second, experiential factor variables showed generally moderate to strong intercorrelations. Notably, AEI Discomfort was unique in exhibiting null to weak correlations with most variables (exception: AEI Reappraisal); and AEI Clarity showed evidence of being nearly interchangeable with RMEQ Mystical and Positive Mood subscales (*r* > .80). Third, correlations between Baseline FFM domains and experiential factor variables were examined. Only AEI Reappraisal and AEI Discomfort showed significant, albeit small, relations with FFM domains (i.e., AEI Reappraisal and Discomfort with Neuroticism [*r* = .22, .22, respectively]). Notably, Openness was not correlated with RMEQ subscales, a result in contrast to previous work^13,14^.

**References**

1. Horn, J. L. A rationale and test for the number of factors in factor analysis. *Psychometrika.* **30**, 179-185 (1965).
2. Velicer, W. F.  Determining the number of components from the matrix of partial correlations. *Psychometrika*. **41***,* 321–327 (1976).
3. DeYoung, C. G., Quilty, L. C., & Peterson, J. B. Between facets and domains: 10 aspects of the Big Five. *Journal of Personality and Social Psychology*. **93**, 880–896 (2007).
4. Judge, T. A., Rodell, J. B., Klinger, R. L., Simon, L. S., & Crawford, E. R. Hierarchical representations of the five-factor model of personality in predicting job performance: Integrating three organizing frameworks with two theoretical perspectives. *Journal of Applied Psychology*. **98,** 875–925 (2013).
5. Hu, L. T., & Bentler, P. M. Cutoff criteria for fit indexes in covariance structure analysis: Conventional criteria versus new alternatives. *Structural Equation Modeling.* **6***,* 1-55 (1999).
6. Vazire, S. Who knows what about a person? The self–other knowledge asymmetry (SOKA) model. *Journal of Personality and Social Psychology*. **98**, 281–300 (2010).
7. Connolly, J. J., Kavanagh, E. J., & Viswesvaran, C. The Convergent Validity between Self and Observer Ratings of Personality: A meta-analytic review. *International Journal of Selection & Assessment*. **15**, 110–117 (2007).
8. Maples, J. L., Guan, A. L., Carter, N., & Miller, J. D. A test of the International Personality Item Pool representation of the Revised NEO Personality Inventory and development of a 120- item IPIP-based measure of the five-factor model. *Psychological Assessment*. **26*,*** 1070–1084 (2014).
9. Goldberg, L. R. et al. The International Personality Item Pool and the future of public-domain personality measures. *Journal of Research in Personality*. **664,** 84–96 (2006).
10. Benjamin, D. J. et al. Redefine statistical significance. *Nature Human Behavior*. **2*,*** 6–10 (2018).
11. Cohen, J. *Statistical Power Analysis for the Behavioral Sciences, 2^nd^ ed.* 24-27 (Lawrence Erlbaum Associates, 1988).
12. van der Linden, D., te Nijenhuis, J., & Bakker, A. B. The General Factor of Personality: A meta-analysis of Big Five intercorrelations and a criterion-related validity study. *Journal of Research in Personality*. **44,** 315–327 (2010).
13. Russ, S. L., Carhart-Harris, R. L., Maruyama, G., & Elliott, M. S. States and traits related to the quality and consequences of psychedelic experiences. *Psychology of Consciousness: Theory, Research, and Practice*. **6,** 1–21 (2019).
14. Studerus, E., Gamma, A., Kometer, M., & Vollenweider, F. X. Prediction of Psilocybin Response in Healthy Volunteers. *Plos One*. **7**, 1–12 (2012).

**Supplementary Tables**

Supplementary Table 1.

Results from Linear Mixed Models

| Predictor / Moderator | Parameter | B | t-value | mR^2^ |
| --- | --- | --- | --- | --- |
| Neuroticism | | | | |
| Main effect of Time (self-report) | |  |  |  |
|  | Intercept | 2.80** | 75.57 | .14 |
|  | Time Post | -.53** | -17.22 |  |
|  | Time FU | -.46** | -14.93 |  |
| Main effect of Time (informant-report) | |  |  |  |
|  | Intercept | 3.03** | 31.55 | .07 |
|  | Time FU | -.34** | -6.52 |  |
| Moderation (self-report) |  |  |  |  |
| Expectancy ΔN | Intercept | 2.42** | 45.14 | .26 |
|  | Expectancy ΔN | .65** | 9.31 |  |
|  | Time Post | -.33** | -7.14 |  |
|  | Time FU | -.25** | -5.33 |  |
|  | Expectancy ΔN X Time Post | -.35** | -5.78 |  |
|  | Expectancy ΔN X Time FU | -.37** | -6.14 |  |
| Expectancy ΔE | Intercept | 2.67** | 57.42 | .16 |
|  | Expectancy ΔE | .34** | 4.54 |  |
|  | Time Post | -.42** | -10.89 |  |
|  | Time FU | -.39** | -10.13 |  |
|  | Expectancy ΔE X Time Post | -.29** | -4.72 |  |
|  | Expectancy ΔE X Time FU | -.19** | -2.96 |  |
| Expectancy ΔO | Intercept | 2.71** | 50.57 | .14 |
|  | Expectancy ΔO | .17* | 2.32 |  |
|  | Time Post | -.45** | -10.04 |  |
|  | Time FU | -.36** | -8.05 |  |
|  | Expectancy ΔO X Time Post | -.17** | -2.72 |  |
|  | Expectancy ΔO X Time FU | -.20** | -3.27 |  |
| Expectancy ΔA | Intercept | 2.70** | 54.41 | .15 |
|  | Expectancy ΔA | .24** | 3.21 |  |
|  | Time Post | -.44** | -10.62 |  |
|  | Time FU | -.36** | -8.73 |  |
|  | Expectancy ΔA X Time Post | -.22** | -3.62 |  |
|  | Expectancy ΔA X Time FU | -.24** | -3.86 |  |
| Expectancy ΔC | Intercept | 2.75** | 54.38 | .14 |
|  | Expectancy ΔC | .12 | 1.62 |  |
|  | Time Post | -.48** | -11.46 |  |
|  | Time FU | -.42** | -9.92 |  |
|  | Expectancy ΔC X Time Post | -.11 | -1.78 |  |
|  | Expectancy ΔC X Time FU | -.10 | -1.57 |  |
| Expectancy Δanxiety | Intercept | 2.57** | 53.06 | .22 |
|  | Expectancy Δanxiety | .50** | 7.08 |  |
|  | Time Post | -.41** | -9.89 |  |
|  | Time FU | -.36** | -8.61 |  |
|  | Mod X Time Post | -.26** | -4.33 |  |
|  | Mod X Time FU | -.23** | -3.70 |  |
| Expectancy Δdepression | Intercept | 2.52** | 49.70 | .22 |
|  | Expectancy Δdep | .56** | 7.87 |  |
|  | Time Post | -.36** | -8.46 |  |
|  | Time FU | -.31** | -7.16 |  |
|  | Expectancy Δdep X Time Post | -.33** | -5.47 |  |
|  | Expectancy Δdep X Time FU | -.30** | -4.98 |  |
| Suggestibility | Intercept | 2.80** | 80.29 | .24 |
|  | Suggestibility | .27** | 7.82 |  |
|  | Time Post | -.53** | -17.55 |  |
|  | Time FU | -.46** | -15.21 |  |
|  | Suggestibility X Time Post | -.10** | -3.25 |  |
|  | Suggestibility X Time FU | -.14** | -4.46 |  |
| Age | Intercept | 2.80** | 75.44 | .14 |
|  | Age | -.06 | -1.57 |  |
|  | Time Post | -.53** | -17.32 |  |
|  | Time FU | -.46** | -15.00 |  |
|  | Age X Time Post | .10** | 3.37 |  |
|  | Age X Time FU | .07* | 2.23 |  |
| Baseline Neuroticism | Intercept | 2.80** | 129.07 | .70 |
|  | Baseline N | .67** | 31.02 |  |
|  | Time Post | -.53** | -20.43 |  |
|  | Time FU | -.46** | -17.72 |  |
|  | Baseline N X Time Post | -.33** | -12.58 |  |
|  | Baseline N X Time FU | -.33** | -12.50 |  |
| Baseline Extraversion | Intercept | 2.80** | 85.12 | .32 |
|  | Baseline E | -.39** | -11.81 |  |
|  | Time Post | -.53** | -18.19 |  |
|  | Time FU | -.46** | -15.77 |  |
|  | Baseline E X Time Post | .21** | 7.26 |  |
|  | Baseline E X Time FU | .18** | 6.11 |  |
| Baseline Conscientiousness | Intercept | 2.80** | 83.63 | .30 |
|  | Baseline C | -.35** | -10.35 |  |
|  | Time Post | -.53** | -17.78 |  |
|  | Time FU | -.46** | -15.42 |  |
|  | Baseline C X Time Post | .17** | 5.63 |  |
|  | Baseline C X Time FU | .14** | 4.53 |  |
| Mystical | Intercept | 2.8** | 73.90 | .16 |
|  | Mystical | .00 | .11 |  |
|  | Time Post | -.52** | -17.22 |  |
|  | Time FU | -.44** | -14.54 |  |
|  | Mystical X Time Post | -.15** | -5.07 |  |
|  | Mystical X Time FU | -.10** | -3.27 |  |
| RMEQ Mystical | Intercept | 2.80** | 74.06 | .16 |
|  | RMEQ Mystical | .02 | .53 |  |
|  | Time Post | -.52** | -17.38 |  |
|  | Time FU | -.44** | -14.67 |  |
|  | RMEQ Mystical X Time Post | -.17** | -5.63 |  |
|  | RMEQ Mystical X Time FU | -.14** | -4.48 |  |
| RMEQ Positivemood | Intercept | 2.80** | 74.21 | .16 |
|  | RMEQ Positivemood | .04 | 1.15 |  |
|  | Time Post | -.52** | -17.64 |  |
|  | Time FU | -.44** | -14.89 |  |
|  | RMEQ Positivemood X Time Post | -.20** | -6.70 |  |
|  | RMEQ Positivemood X Time FU | -.16** | -5.45 |  |
| RMEQ Timespace | Intercept | 2.80** | 73.91 | .16 |
|  | RMEQ Timespace | -.01 | -.22 |  |
|  | Time Post | -.52** | -17.11 |  |
|  | Time FU | -.44** | -14.45 |  |
|  | RMEQ Timespace X Time Post | -.11** | -3.54 |  |
|  | RMEQ Timespace X Time FU | -.13** | -4.14 |  |
| RMEQ Ineffable | Intercept | 2.80** | 72.94 | .14 |
|  | RMEQ Ineffable | .06 | 1.52 |  |
|  | Time Post | -.52** | -16.94 |  |
|  | Time FU | -.44** | -14.31 |  |
|  | RMEQ Ineffable X Time Post | -.08* | -2.52 |  |
|  | RMEQ Ineffable X Time FU | -.09** | -3.02 |  |
| Ego Dissolution | Intercept | 2.8** | 73.52 | .15 |
|  | Ego Dissolution | .03 | .75 |  |
|  | Time Post | -.52** | -17.20 |  |
|  | Time FU | -.44** | -14.52 |  |
|  | Ego Dissolution X Time Post | -.15** | -4.80 |  |
|  | Ego Dissolution X Time FU | -.11** | -3.62 |  |
| AEI Clarity | Intercept | 2.84** | 66.25 | .17 |
|  | AEI Clarity | .02 | .40 |  |
|  | Time Post | -.51** | -15.69 |  |
|  | Time FU | -.47** | -14.25 |  |
|  | AEI Clarity X Time Post | -.19** | -5.78 |  |
|  | AEI Clarity X Time FU | -.18** | -5.54 |  |
| AEI Reappraisal | Intercept | 2.84** | 65.35 | .15 |
|  | AEI Reappraisal | .15** | 3.42 |  |
|  | Time Post | -.51** | -15.89 |  |
|  | Time FU | -.47** | -14.42 |  |
|  | AEI Reappraisal X Time Post | -.20** | -6.21 |  |
|  | AEI Reappraisal X Time FU | -.21** | -6.40 |  |
| Trusted shaman | Intercept | 2.82** | 65.64 | .13 |
|  | Trusted shaman | .07 | 1.54 |  |
|  | Time Post | -.51** | -15.33 |  |
|  | Time FU | -.46** | -13.88 |  |
|  | Trusted shaman X Time Post | -.12** | -3.54 |  |
|  | Trusted shaman X Time FU | -.13** | -3.84 |  |
| Icaro prayer healing | Intercept | 2.82** | 65.76 | .14 |
|  | Icaro prayer healing | .06 | 1.49 |  |
|  | Time Post | -.51** | -15.42 |  |
|  | Time FU | -.46** | -13.96 |  |
|  | Icaro prayer healing X Time Post | -.13** | -4.10 |  |
|  | Icaro prayer healing X Time FU | -.14** | -4.18 |  |
| Medicine cleaning | Intercept | 2.82** | 65.79 | .14 |
|  | Medicine cleaning | .09* | 2.19 |  |
|  | Time Post | -.51** | -15.75 |  |
|  | Time FU | -.46** | -14.28 |  |
|  | Medicine cleaning X Time Post | -.17** | -5.30 |  |
|  | Medicine cleaning X Time FU | -.16** | -4.83 |  |
| Purging self | Intercept | 2.82** | 66.42 | .15 |
|  | Purging self | .19** | 4.43 |  |
|  | Time Post | -.51** | -15.94 |  |
|  | Time FU | -.46** | -14.43 |  |
|  | Purging self X Time Post | -.20** | -6.20 |  |
|  | Purging self X Time FU | -.20** | -6.15 |  |
| Purged physical ailment | Intercept | 2.82** | 65.48 | .13 |
|  | Purged physical ailment | .03 | .76 |  |
|  | Time Post | -.51** | -15.29 |  |
|  | Time FU | -.46** | -13.86 |  |
|  | Purged physical ailment X Time Post | -.10** | -2.95 |  |
|  | Purged physical ailment X Time FU | -.09** | -2.63 |  |
| Purged completely | Intercept | 2.83** | 66.32 | .15 |
|  | Purged completely | .06 | 1.46 |  |
|  | Time Post | -.51** | -15.65 |  |
|  | Time FU | -.46** | -14.26 |  |
|  | Purged completely X Time Post | -.18** | -5.53 |  |
|  | Purged completely X Time FU | -.14** | -4.46 |  |
| Relationship object | Intercept | 2.82** | 65.38 | .13 |
|  | Relationship object | .05 | 1.27 |  |
|  | Time Post | -.51** | -15.27 |  |
|  | Time FU | -.46** | -13.84 |  |
|  | Relationship object X Time Post | -.10** | -3.06 |  |
|  | Relationship object X Time FU | -.06 | -1.81 |  |
| Extraversion | | | | |
| Main effect of Time (self-report) | |  |  |  |
|  | Intercept | 3.37** | 104.34 | .04 |
|  | Time Post | .23** | 9.98 |  |
|  | Time FU | .20** | 8.57 |  |
| Main effect of Time (informant-report) | |  |  |  |
|  | Intercept | 3.53** | 42.42 | .00 |
|  | Time FU | .08 | 1.87 |  |
| Moderation (self-report) |  |  |  |  |
| Expectancy ΔN | Intercept | 3.60** | 74.76 | .12 |
|  | Expectancy ΔN | -.4** | -6.31 |  |
|  | Time Post | .14** | 3.82 |  |
|  | Time FU | .11** | 2.99 |  |
|  | Expectancy ΔN X Time Post | .16** | 3.52 |  |
|  | Expectancy ΔN X Time FU | .16** | 3.40 |  |
| Expectancy ΔE | Intercept | 3.50** | 86.66 | .08 |
|  | Expectancy ΔE | -.33** | -5.05 |  |
|  | Time Post | .14** | 4.90 |  |
|  | Time FU | .13** | 4.46 |  |
|  | Expectancy ΔE X Time Post | .24** | 5.09 |  |
|  | Expectancy ΔE X Time FU | .18** | 3.96 |  |
| Expectancy Δdepression | Intercept | 3.54** | 78.44 | .09 |
|  | Expectancy Δdep | -.33** | -5.33 |  |
|  | Time Post | .14** | 4.38 |  |
|  | Time FU | .13** | 3.97 |  |
|  | Expectancy Δdep X Time Post | .17** | 3.70 |  |
|  | Expectancy Δdep X Time FU | .13** | 2.88 |  |
| Sex | Intercept | 3.34** | 82.49 | .04 |
|  | Sex | .08 | 1.24 |  |
|  | Time Post | .29** | 10.08 |  |
|  | Time FU | .24** | 8.34 |  |
|  | Sex X Time Post | -.16** | -3.43 |  |
|  | Sex X Time FU | -.12* | -2.47 |  |
| Age | Intercept | 3.37** | 106.26 | .07 |
|  | Age | -.04 | -1.25 |  |
|  | Time Post | .23** | 10.09 |  |
|  | Time FU | .20** | 8.63 |  |
|  | Age X Time Post | -.09** | -3.82 |  |
|  | Age X Time FU | -.05 | -2.05 |  |
| Baseline Neuroticism | Intercept | 3.37** | 118.97 | .26 |
|  | Baseline N | -.34** | -11.97 |  |
|  | Time Post | .23** | 10.56 |  |
|  | Time FU | .20** | 9.07 |  |
|  | Baseline N X Time Post | .15** | 6.68 |  |
|  | Baseline N X Time FU | .15** | 7.01 |  |
| Baseline Extraversion | Intercept | 3.37** | 206.65 | .75 |
|  | Baseline E | .59** | 36.02 |  |
|  | Time Post | .23** | 11.63 |  |
|  | Time FU | .20** | 9.99 |  |
|  | Baseline E X Time Post | -.24** | -12.04 |  |
|  | Baseline E X Time FU | -.23** | -11.45 |  |
| Baseline Conscientiousness | Intercept | 3.37** | 109.90 | .14 |
|  | Baseline C | .22** | 7.17 |  |
|  | Time Post | .23** | 10.18 |  |
|  | Time FU | .20** | 8.74 |  |
|  | Baseline C X Time Post | -.08** | -3.65 |  |
|  | Baseline C X Time FU | -.10** | -4.40 |  |
| Mystical | Intercept | 3.36** | 100.40 | .06 |
|  | Mystical | .03 | .81 |  |
|  | Time Post | .22** | 9.61 |  |
|  | Time FU | .19** | 8.19 |  |
|  | Mystical X Time Post | .10** | 4.11 |  |
|  | Mystical X Time FU | .04 | 1.53 |  |
| RMEQ Mystical | Intercept | 3.36** | 100.06 | .05 |
|  | RMEQ Mystical | .00 | .08 |  |
|  | Time Post | .22** | 9.61 |  |
|  | Time FU | .19** | 8.20 |  |
|  | RMEQ Mystical X Time Post | .09** | 4.08 |  |
|  | RMEQ Mystical X Time FU | .07** | 2.86 |  |
| RMEQ Positivemood | Intercept | 3.36** | 100.06 | .05 |
|  | RMEQ Positivemood | -.01 | -.20 |  |
|  | Time Post | .22** | 9.66 |  |
|  | Time FU | .19** | 8.24 |  |
|  | RMEQ Positivemood X Time Post | .11** | 4.62 |  |
|  | RMEQ Positivemood X Time FU | .07** | 3.09 |  |
| Ego Dissolution | Intercept | 3.36** | 99.55 | .04 |
|  | Ego Dissolution | -.02 | -.64 |  |
|  | Time Post | .22** | 9.58 |  |
|  | Time FU | .19** | 8.17 |  |
|  | Ego Dissolution X Time Post | .09** | 3.70 |  |
|  | Ego Dissolution X Time FU | .06* | 2.58 |  |
| AEI Clarity | Intercept | 3.32** | 87.72 | .07 |
|  | AEI Clarity | .02 | .41 |  |
|  | Time Post | .22** | 8.48 |  |
|  | Time FU | .22** | 8.43 |  |
|  | AEI Clarity X Time Post | .12** | 4.84 |  |
|  | AEI Clarity X Time FU | .09** | 3.40 |  |
| AEI Reappraisal | Intercept | 3.32** | 86.78 | .05 |
|  | AEI Reappraisal | -.07 | -1.89 |  |
|  | Time Post | .22** | 8.62 |  |
|  | Time FU | .22** | 8.57 |  |
|  | AEI Reappraisal X Time Post | .15** | 5.86 |  |
|  | AEI Reappraisal X Time FU | .12** | 4.62 |  |
| AEI Discomfort | Intercept | 3.32** | 86.81 | .05 |
|  | AEI Discomfort | -.11** | -2.96 |  |
|  | Time Post | .22** | 8.42 |  |
|  | Time FU | .22** | 8.37 |  |
|  | AEI Discomfort X Time Post | .09** | 3.58 |  |
|  | AEI Discomfort X Time FU | .10** | 4.06 |  |
| Icaro prayer healing | Intercept | 3.33** | 88.14 | .05 |
|  | Icaro prayer healing | -.01 | -.36 |  |
|  | Time Post | .21** | 8.45 |  |
|  | Time FU | .21** | 8.38 |  |
|  | Icaro prayer healing X Time Post | .08** | 3.25 |  |
|  | Icaro prayer healing X Time FU | .08** | 3.06 |  |
| Medicine cleaning | Intercept | 3.33** | 87.80 | .05 |
|  | Medicine cleaning | -.05 | -1.20 |  |
|  | Time Post | .22** | 8.61 |  |
|  | Time FU | .21** | 8.55 |  |
|  | Medicine cleaning X Time Post | .11** | 4.20 |  |
|  | Medicine cleaning X Time FU | .11** | 4.26 |  |
| Purging self | Intercept | 3.33** | 88.55 | .05 |
|  | Purging self | -.13** | -3.40 |  |
|  | Time Post | .21** | 8.60 |  |
|  | Time FU | .21** | 8.53 |  |
|  | Purging self X Time Post | .11** | 4.51 |  |
|  | Purging self X Time FU | .11** | 4.56 |  |
| Purged completely | Intercept | 3.33** | 88.19 | .04 |
|  | Purged completely | -.07 | -1.79 |  |
|  | Time Post | .22** | 8.59 |  |
|  | Time FU | .21** | 8.58 |  |
|  | Purged completely X Time Post | .12** | 4.83 |  |
|  | Purged completely X Time FU | .09** | 3.56 |  |
| Openness | | | | |
| Main effect of Time (self-report) | |  |  |  |
|  | Intercept | 3.77** | 150.91 | .00 |
|  | Time Post | .06** | 3.68 |  |
|  | Time FU | .04** | 2.72 |  |
| Main effect of Time (informant-report) | |  |  |  |
|  | Intercept | 3.68 | 49.72 | .01 |
|  | Time FU | .09* | 2.15 |  |
| Moderation (self-report) |  |  |  |  |
| Age | Intercept | 3.77 | 152.79 | .04 |
|  | Age | -.05 | -1.90 |  |
|  | Time Post | .06** | 3.68 |  |
|  | Time FU | .04** | 2.71 |  |
|  | Age X Time Post | -.05** | -3.16 |  |
|  | Age X Time FU | -.02 | -1.42 |  |
| Baseline Openness | Intercept | 3.77** | 296.70 | .74 |
|  | Baseline O | .42** | 32.96 |  |
|  | Time Post | .06** | 3.95 |  |
|  | Time FU | .04** | 2.92 |  |
|  | Baseline O X Time Post | -.11** | -7.46 |  |
|  | Baseline O X Time FU | -.13** | -8.18 |  |
| RMEQ Mystical | Intercept | 3.78** | 147.91 | .04 |
|  | RMEQ Mystical | .05* | 2.10 |  |
|  | Time Post | .05** | 3.05 |  |
|  | Time FU | .04* | 2.19 |  |
|  | RMEQ Mystical X Time Post | .04* | 2.54 |  |
|  | RMEQ Mystical X Time FU | .02 | 1.35 |  |
| RMEQ Positive Mood | Intercept | 3.78** | 147.71 | .04 |
|  | RMEQ Positive Mood | .05 | 1.83 |  |
|  | Time Post | .05** | 3.06 |  |
|  | Time FU | .04* | 2.20 |  |
|  | RMEQ Positive Mood X Time Post | .05** | 3.01 |  |
|  | RMEQ Positive Mood X Time FU | .02 | 1.42 |  |
| AEI Clarity | Intercept | 3.77** | 128.55 | .04 |
|  | AEI Clarity | .05 | 1.55 |  |
|  | Time Post | .05** | 2.64 |  |
|  | Time FU | .05* | 2.57 |  |
|  | AEI Clarity X Time Post | .05** | 2.89 |  |
|  | AEI Clarity X Time FU | .04* | 2.43 |  |
| Agreeableness | | | | |
| Main effect of Time (self-report) | |  |  |  |
|  | Intercept | 3.63** | 155.92 | .03 |
|  | Time Post | .14** | 8.70 |  |
|  | Time FU | .11** | 6.46 |  |
| Main effect of Time (informant-report) | |  |  |  |
|  | Intercept | 3.72** | 48.28 | .00 |
|  | Time FU | .07 | 1.67 |  |
| Moderation (self-report) |  |  |  |  |
| Lifetime Ayahuasca use | Intercept | 3.6** | 128.82 | .03 |
|  | Lifetime Ayahuasca use | .15* | 2.30 |  |
|  | Time Post | .16** | 8.43 |  |
|  | Time FU | .12** | 6.35 |  |
|  | Lifetime Ayahuasca use X Time Post | -.13** | -2.98 |  |
|  | Lifetime Ayahuasca use X Time FU | -.10* | -2.36 |  |
| Baseline Openness | Intercept | 3.63** | 161.09 | .09 |
|  | Baseline O | .12** | 5.51 |  |
|  | Time Post | .14** | 8.79 |  |
|  | Time FU | .11** | 6.53 |  |
|  | Baseline O X Time Post | -.04* | -2.13 |  |
|  | Baseline O X Time FU | -.06** | -3.54 |  |
| Baseline Agreeableness | Intercept | 3.63** | 295.75 | .73 |
|  | Baseline A | .4** | 32.69 |  |
|  | Time Post | .14** | 9.59 |  |
|  | Time FU | .11** | 7.12 |  |
|  | Baseline A X Time Post | -.14** | -9.23 |  |
|  | Baseline A X Time FU | -.14** | -9.03 |  |
| Baseline Conscientiousness | Intercept | 3.63** | 158.47 | .06 |
|  | Baseline C | .1** | 4.25 |  |
|  | Time Post | .14** | 8.77 |  |
|  | Time FU | .11** | 6.51 |  |
|  | Baseline C X Time Post | -.05** | -2.78 |  |
|  | Baseline C X Time FU | -.05** | -2.75 |  |
| Mystical | Intercept | 3.63** | 150.23 | .04 |
|  | Mystical | .00 | .06 |  |
|  | Time Post | .14** | 8.24 |  |
|  | Time FU | .11** | 6.25 |  |
|  | Mystical X Time Post | .06** | 3.69 |  |
|  | Mystical X Time FU | .03 | 1.95 |  |
| RMEQ Mystical | Intercept | 3.63** | 150.03 | .03 |
|  | RMEQ Mystical | -.02 | -.76 |  |
|  | Time Post | .14** | 8.28 |  |
|  | Time FU | .11** | 6.28 |  |
|  | RMEQ Mystical X Time Post | .07** | 4.18 |  |
|  | RMEQ Mystical X Time FU | .05** | 3.11 |  |
| RMEQ Positivemood | Intercept | 3.63** | 150.19 | .04 |
|  | RMEQ Positivemood | -.01 | -.43 |  |
|  | Time Post | .14** | 8.28 |  |
|  | Time FU | .11** | 6.28 |  |
|  | RMEQ Positivemood X Time Post | .07** | 4.19 |  |
|  | RMEQ Positivemood X Time FU | .05** | 2.76 |  |
| Ego Dissolution | Intercept | 3.63** | 150.15 | .04 |
|  | Ego Dissolution | -.02 | -.65 |  |
|  | Time Post | .14** | 8.29 |  |
|  | Time FU | .11** | 6.28 |  |
|  | Ego Dissolution X Time Post | .07** | 4.03 |  |
|  | Ego Dissolution X Time FU | .06** | 3.52 |  |
| AEI Clarity | Intercept | 3.62** | 128.20 | .03 |
|  | AEI Clarity | -.06* | -2.06 |  |
|  | Time Post | .13** | 6.81 |  |
|  | Time FU | .11** | 5.68 |  |
|  | AEI Clarity X Time Post | .08** | 3.96 |  |
|  | AEI Clarity X Time FU | .06** | 2.98 |  |
| AEI Reappraisal | Intercept | 3.62** | 127.97 | .03 |
|  | AEI Reappraisal | -.03 | -1.09 |  |
|  | Time Post | .13** | 6.76 |  |
|  | Time FU | .11** | 5.64 |  |
|  | AEI Reappraisal X Time Post | .06** | 3.38 |  |
|  | AEI Reappraisal X Time FU | .03 | 1.78 |  |
| Purging self | Intercept | 3.62** | 132.15 | .04 |
|  | Purging self | .01 | .27 |  |
|  | Time Post | .13** | 6.93 |  |
|  | Time FU | .10** | 5.55 |  |
|  | Purging self X Time Post | .06** | 3.19 |  |
|  | Purging self X Time FU | .04* | 2.19 |  |
| Conscientiousness | | | | |
| Main effect of Time (self-report) | |  |  |  |
|  | Intercept | 3.56** | 135.08 | .05 |
|  | Time Post | .23** | 11.48 |  |
|  | Time FU | .17** | 8.28 |  |
| Main effect of Time (informant-report) | |  |  |  |
|  | Intercept | 3.61** | 43.80 | .01 |
|  | Time FU | .10* | 2.24 |  |
| Moderation (self-report) |  |  |  |  |
| Expectancy ΔN | Intercept | 3.69** | 91.26 | .08 |
|  | Expectancy ΔN | -.22** | -4.20 |  |
|  | Time Post | .17** | 5.49 |  |
|  | Time FU | .06* | 2.07 |  |
|  | Expectancy ΔN X Time Post | .11** | 2.64 |  |
|  | Expectancy ΔN X Time FU | .18** | 4.37 |  |
| Expectancy ΔE | Intercept | 3.64** | 109.12 | .07 |
|  | Expectancy ΔE | -.19** | -3.53 |  |
|  | Time Post | .19** | 7.34 |  |
|  | Time FU | .11** | 4.47 |  |
|  | Expectancy ΔE X Time Post | .12** | 2.88 |  |
|  | Expectancy ΔE X Time FU | .14** | 3.39 |  |
| Expectancy ΔC | Intercept | 3.66** | 103.05 | .08 |
|  | Expectancy ΔC | -.21** | -4.04 |  |
|  | Time Post | .18** | 6.70 |  |
|  | Time FU | .09** | 3.23 |  |
|  | Expectancy ΔC X Time Post | .11** | 2.75 |  |
|  | Expectancy ΔC X Time FU | .17** | 4.33 |  |
| Age | Intercept | 3.57** | 135.81 | .06 |
|  | Age | .07** | 2.70 |  |
|  | Time Post | .24** | 11.63 |  |
|  | Time FU | .17** | 8.36 |  |
|  | Age X Time Post | -.07** | -3.68 |  |
|  | Age X Time FU | -.05* | -2.33 |  |
| Baseline Neuroticism | Intercept | 3.56** | 150.02 | .23 |
|  | Baseline N | -.24** | -9.90 |  |
|  | Time Post | .23** | 11.73 |  |
|  | Time FU | .17** | 8.46 |  |
|  | Baseline N X Time Post | .07** | 3.41 |  |
|  | Baseline N X Time FU | .10** | 4.83 |  |
| Baseline Extraversion | Intercept | 3.56** | 142.61 | .15 |
|  | Baseline E | .17** | 6.84 |  |
|  | Time Post | .23** | 11.61 |  |
|  | Time FU | .17** | 8.37 |  |
|  | Baseline E X Time Post | -.04 | -1.84 |  |
|  | Baseline E X Time FU | -.07** | -3.66 |  |
| Baseline Conscientiousness | Intercept | 3.56** | 239.43 | .70 |
|  | Baseline C | .46** | 30.69 |  |
|  | Time Post | .23** | 12.82 |  |
|  | Time FU | .17** | 9.24 |  |
|  | Baseline C X Time Post | -.17** | -9.32 |  |
|  | Baseline C X Time FU | -.19** | -10.21 |  |
| Mystical | Intercept | 3.55** | 131.48 | .06 |
|  | Mystical | .00 | -.05 |  |
|  | Time Post | .23** | 11.10 |  |
|  | Time FU | .17** | 8.07 |  |
|  | Mystical X Time Post | .07** | 3.42 |  |
|  | Mystical X Time FU | .04 | 1.71 |  |
| RMEQ Mystical | Intercept | 3.55** | 131.79 | .07 |
|  | RMEQ Mystical | .01 | .34 |  |
|  | Time Post | .23** | 11.08 |  |
|  | Time FU | .17** | 8.05 |  |
|  | RMEQ Mystical X Time Post | .07** | 3.12 |  |
|  | RMEQ Mystical X Time FU | .04* | 2.11 |  |
| RMEQ Positivemood | Intercept | 3.55** | 131.62 | .06 |
|  | RMEQ Positivemood | .00 | -.01 |  |
|  | Time Post | .23** | 11.10 |  |
|  | Time FU | .17** | 8.06 |  |
|  | RMEQ Positivemood X Time Post | .07** | 3.26 |  |
|  | RMEQ Positivemood X Time FU | .05 | 2.43 |  |
| Ego Dissolution | Intercept | 3.55** | 131.17 | .06 |
|  | Ego Dissolution | -.05 | -1.70 |  |
|  | Time Post | .23** | 11.16 |  |
|  | Time FU | .17** | 8.11 |  |
|  | Ego Dissolution X Time Post | .08** | 4.07 |  |
|  | Ego Dissolution X Time FU | .05* | 2.34 |  |
| AEI Clarity | Intercept | 3.55** | 115.99 | .06 |
|  | AEI Clarity | -.01 | -.36 |  |
|  | Time Post | .22** | 9.83 |  |
|  | Time FU | .18** | 8.07 |  |
|  | AEI Clarity X Time Post | .08** | 3.68 |  |
|  | AEI Clarity X Time FU | .08** | 3.38 |  |
| AEI Reappraisal | Intercept | 3.55** | 115.43 | .06 |
|  | AEI Reappraisal | -.06* | -1.98 |  |
|  | Time Post | .22** | 9.82 |  |
|  | Time FU | .18** | 8.06 |  |
|  | AEI Reappraisal X Time Post | .07** | 3.28 |  |
|  | AEI Reappraisal X Time FU | .08** | 3.60 |  |
| Medicine cleaning | Intercept | 3.55** | 117.26 | .05 |
|  | Medicine cleaning | -.04 | -1.35 |  |
|  | Time Post | .22** | 10.06 |  |
|  | Time FU | .18** | 8.11 |  |
|  | Medicine cleaning X Time Post | .05* | 2.42 |  |
|  | Medicine cleaning X Time FU | .07** | 3.30 |  |
| Purging self | Intercept | 3.55** | 118.83 | .07 |
|  | Purging self | -.10** | -3.33 |  |
|  | Time Post | .22** | 10.00 |  |
|  | Time FU | .18** | 8.06 |  |
|  | Purging self X Time Post | .06** | 2.92 |  |
|  | Purging self X Time FU | .08** | 3.44 |  |
| Note. FU = Follow-up. RMEQ = Revised Mystical Experience Questionnaire. AEI = Ayahuasca Experience Inventory. mR^2^ = marginal R^2^ indicating degree to which fixed effect variables account for variance in personality. | | | | |

Supplementary Table 2.

Internal Consistency (α) of personality domains and facets

|  | Self-report | | |  | Informant-report | |
| --- | --- | --- | --- | --- | --- | --- |
| Domain/Facet | T1 | T2 | T3 |  | T1 | T3 |
| Neuroticism | .93 | .92 | .92 |  | .85 | .86 |
| Extraversion | .91 | .90 | .89 |  | .87 | .84 |
| Openness | .80 | .81 | .79 |  | .78 | .71 |
| Agreeableness | .80 | .81 | .80 |  | .87 | .85 |
| Conscientiousness | .86 | .87 | .85 |  | .86 | .87 |
| N1 Anxiety | .82 | .80 | .79 |  | .82 | .83 |
| N2 Anger | .89 | .80 | .85 |  | .88 | .89 |
| N3 Depression | .89 | .85 | .87 |  | .79 | .86 |
| N4 Self-Consciousness | .75 | .75 | .71 |  | .71 | .61 |
| N5 Immoderation | .79 | .81 | .78 |  | .80 | .80 |
| N6 Vulnerability | .76 | .74 | .74 |  | .90 | .87 |
| E1 Friendliness | .85 | .80 | .78 |  | .85 | .75 |
| E2 Gregariousness | .86 | .82 | .84 |  | .86 | .83 |
| E3 Assertiveness | .84 | .82 | .74 |  | .86 | .88 |
| E4 Activity Level | .74 | .75 | .73 |  | .83 | .85 |
| E5 Excitement-Seeking | .71 | .66 | .72 |  | .77 | .79 |
| E6 Cheerfulness | .78 | .79 | .75 |  | .89 | .85 |
| O1 Imagination | .79 | .78 | .73 |  | .73 | .67 |
| O2 Artistic Interests | .76 | .73 | .73 |  | .92 | .88 |
| O3 Emotionality | .80 | .72 | .75 |  | .71 | .67 |
| O4 Adventurousness | .81 | .75 | .76 |  | .74 | .74 |
| O5 Intellect | .81 | .80 | .77 |  | .85 | .84 |
| O6 Liberalism | .68 | .59 | .61 |  | .34 | .42 |
| A1 Trust | .87 | .84 | .85 |  | .86 | .84 |
| A2 Morality | .65 | .64 | .60 |  | .82 | .85 |
| A3 Altruism | .63 | .65 | .57 |  | .84 | .85 |
| A4 Cooperation | .62 | .66 | .67 |  | .81 | .82 |
| A5 Modesty | .70 | .63 | .63 |  | .67 | .57 |
| A6 Sympathy | .63 | .61 | .66 |  | .88 | .86 |
| C1 Self-efficacy | .78 | .82 | .73 |  | .81 | .79 |
| C2 Orderliness | .73 | .68 | .73 |  | .87 | .84 |
| C3 Dutifulness | .66 | .67 | .61 |  | .85 | .70 |
| C4 Achievement-Striving | .66 | .64 | .61 |  | .75 | .80 |
| C5 Self-Discipline | .87 | .85 | .84 |  | .76 | .75 |
| C6 Cautiousness | .85 | .79 | .84 |  | .88 | .86 |
| *Note*. Values represent Cronbach alphas (α); T1 = Baseline;  T2 = Post; T3 = Follow-up. | | | | | | |

Supplementary Table 3.

Model fit indices for Higher-order Acute Experience factors

| Structure | MAP | VSS | RMSEA | TLI | SRMR | BIC |
| --- | --- | --- | --- | --- | --- | --- |
| One-factor | .07 | .89 | .19 | .81 | .08 | 86 |
| Two-factor | .08 | .91 | .15 | .88 | .04 | 16 |
| *Note.* MAP = Velicer's Minimum Average Partial index; VSS = Very Simple Structure; RMSEA = root mean square error of approximation index; TLI = Tucker-Lewis index; SRMR = Standardized Root Mean Square Residual; BIC = Bayesian Information Criterion | | | | | | |

Supplementary Table 4.

Exploratory factor analysis of experiential factor variables

| Item | One | Two |
| --- | --- | --- |
| RMEQ Mystical | **.97** | -.15 |
| RMEQ Positive Mood | **.90** | -.19 |
| RMEQ Timespace | **.64** | .16 |
| RMEQ Ineffable | **.50** | .18 |
| AEI Clarity | **.98** | -.18 |
| AEI Reappraisal | **.68** | .20 |
| AEI Discomfort | -.12 | **.98** |
| Ego Dissolution Inventory | **.72** | -.01 |
| *Note.* Strongest loading for each item is in bold. | | |

Supplementary Table 5.

Correlations between Self- and Informant-report personality domains

|  | Self-report | | | | |
| --- | --- | --- | --- | --- | --- |
| Informant-report | N (T1, T3) | E (T1, T3) | O (T1, T3) | A (T1, T3) | C (T1, T3) |
| Neuroticism (T1, T3) | .52*, .41* |  |  |  |  |
| Extraversion (T1, T3) |  | .66*, .61* |  |  |  |
| Openness (T1, T3) |  |  | .53*, .33* |  |  |
| Agreeableness (T1, T3) |  |  |  | .38*, .31* |  |
| Conscientiousness (T1, T3) |  |  |  |  | .55*, .35* |
| *Note*. N = Neuroticism; E = Extraversion; O = Openness; A = Agreeableness; C = Conscientiousness;  T1 = Baseline; T3 = Follow-up; N = 91; **p* < .005. | | | | | |

Supplementary Table 6.

Domain and Facet means compared to Undergraduate and Community population

|  | Undergraduate  (N = 359) | Community  (N = 501) | Baseline  Self | Post  Self | Follow-up  Self | Baseline  Informant | Follow-up  Informant |
| --- | --- | --- | --- | --- | --- | --- | --- |
| Neuroticism | 2.62 | 2.34 | 2.80 | 2.27 | 2.34 | 2.68 | 2.34 |
| Extraversion | 3.78 | 3.39 | 3.37 | 3.60 | 3.57 | 3.61 | 3.69 |
| Openness | 3.33 | 3.26 | 3.77 | 3.83 | 3.81 | 3.76 | 3.85 |
| Agreeableness | 3.70 | 3.89 | 3.63 | 3.77 | 3.73 | 3.79 | 3.85 |
| Conscientiousness | 3.53 | 3.97 | 3.56 | 3.80 | 3.73 | 3.71 | 3.81 |
| N1 Anxiety | 3.06 | 2.46 | 2.98 | 2.40 | 2.49 | 3.29 | 2.76 |
| N2 Anger | 2.62 | 2.52 | 2.85 | 2.30 | 2.35 | 2.52 | 2.11 |
| N3 Depression | 2.18 | 2.06 | 2.71 | 2.01 | 2.09 | 2.72 | 2.22 |
| N4 Self-Conscious | 2.56 | 2.46 | 2.70 | 2.24 | 2.28 | 2.23 | 1.86 |
| N5 Immoderation | 2.95 | 2.42 | 3.09 | 2.61 | 2.74 | 2.80 | 2.72 |
| N6 Vulnerability | 2.37 | 2.13 | 2.50 | 2.06 | 2.09 | 2.53 | 2.35 |
| E1 Friendliness | 4.01 | 3.69 | 3.42 | 3.81 | 3.76 | 3.86 | 3.98 |
| E2 Gregariousness | 3.57 | 2.71 | 2.71 | 3.00 | 2.91 | 3.12 | 3.12 |
| E3 Assertiveness | 3.57 | 3.41 | 3.50 | 3.64 | 3.68 | 3.54 | 3.68 |
| E4 Activity Level | 3.55 | 3.61 | 3.28 | 3.41 | 3.39 | 3.31 | 3.41 |
| E5 Excite-Seeking | 3.83 | 3.11 | 3.70 | 3.66 | 3.68 | 4.02 | 4.03 |
| E6 Cheerfulness | 4.14 | 3.83 | 3.60 | 4.08 | 3.98 | 3.79 | 3.90 |
| O1 Imagination | 3.71 | 3.16 | 3.87 | 3.85 | 3.87 | 3.55 | 3.74 |
| O2 Artistic Interest | 3.63 | 3.83 | 3.91 | 4.11 | 4.12 | 4.09 | 4.18 |
| O3 Emotionality | 3.40 | 3.32 | 3.51 | 3.45 | 3.40 | 3.56 | 3.58 |
| O4 Adventurous | 2.97 | 2.87 | 3.50 | 3.74 | 3.67 | 3.41 | 3.59 |
| O5 Intellect | 3.70 | 3.53 | 4.17 | 4.21 | 4.20 | 4.11 | 4.17 |
| O6 Liberalism | 2.58 | 2.85 | 3.65 | 3.61 | 3.63 | 3.86 | 3.85 |
| A1 Trust | 3.65 | 3.67 | 3.29 | 3.64 | 3.58 | 3.41 | 3.43 |
| A2 Morality | 3.52 | 4.12 | 3.55 | 3.73 | 3.67 | 4.25 | 4.35 |
| A3 Altruism | 4.24 | 4.22 | 4.03 | 4.20 | 4.14 | 4.01 | 3.99 |
| A4 Cooperation | 3.79 | 4.37 | 3.89 | 4.17 | 4.13 | 4.01 | 4.21 |
| A5 Modesty | 3.20 | 3.21 | 3.24 | 3.04 | 3.10 | 3.31 | 3.35 |
| A6 Sympathy | 3.77 | 3.74 | 3.76 | 3.83 | 3.79 | 3.74 | 3.79 |
| C1 Self-efficacy | 3.93 | 4.02 | 3.85 | 4.01 | 3.98 | 3.89 | 3.94 |
| C2 Orderliness | 3.24 | 3.86 | 3.38 | 3.60 | 3.55 | 3.09 | 3.39 |
| C3 Dutifulness | 4.14 | 4.51 | 4.01 | 4.18 | 4.14 | 4.03 | 4.14 |
| C4 Achieve-Strive | 4.00 | 4.00 | 3.88 | 3.97 | 3.94 | 4.16 | 4.09 |
| C5 Self-Discipline | 2.75 | 3.59 | 3.02 | 3.43 | 3.31 | 3.66 | 3.75 |
| C6 Cautiousness | 3.14 | 3.84 | 3.26 | 3.60 | 3.48 | 3.44 | 3.54 |
| *Note*. Self = Self-report; Informant = Informant-report. | | | | | | | |

Supplementary Table 7.

Domain and Facet standard deviation compared to Undergraduate and Community population

|  | Undergraduate  (N = 359) | Community  (N = 501) | Baseline  Self | Post  Self | Follow-up  Self | Baseline  Informant | Follow-up  Informant |
| --- | --- | --- | --- | --- | --- | --- | --- |
| Neuroticism | .58 | .57 | .68 | .54 | .55 | .67 | .58 |
| Extraversion | .54 | .51 | .59 | .48 | .47 | .64 | .57 |
| Openness | .60 | .57 | .42 | .39 | .38 | .53 | .43 |
| Agreeableness | .51 | .39 | .40 | .36 | .36 | .60 | .52 |
| Conscientiousness | .51 | .44 | .46 | .41 | .40 | .61 | .56 |
| N1 Anxiety | .96 | .81 | .92 | .78 | .75 | 1.03 | .92 |
| N2 Anger | .97 | .88 | .96 | .72 | .76 | 1.04 | .82 |
| N3 Depression | .91 | .88 | 1.00 | .72 | .75 | 1.03 | .86 |
| N4 Self-Conscious | .83 | .76 | .85 | .66 | .67 | 1.01 | .75 |
| N5 Immoderation | .85 | .86 | .88 | .81 | .81 | .96 | .93 |
| N6 Vulnerability | .76 | .69 | .79 | .59 | .59 | .98 | .81 |
| E1 Friendliness | .73 | .81 | .88 | .64 | .64 | .94 | .76 |
| E2 Gregariousness | .98 | .93 | .96 | .86 | .86 | 1.12 | 1.07 |
| E3 Assertiveness | .86 | .80 | .77 | .68 | .61 | .90 | .84 |
| E4 Activity Level | .71 | .82 | .79 | .71 | .72 | .93 | .93 |
| E5 Excite-Seeking | .75 | .75 | .66 | .57 | .59 | .82 | .73 |
| E6 Cheerfulness | .62 | .70 | .80 | .63 | .62 | .92 | .81 |
| O1 Imagination | .88 | .85 | .76 | .68 | .67 | .87 | .74 |
| O2 Artistic Interest | .98 | .83 | .74 | .67 | .65 | .87 | .82 |
| O3 Emotionality | .88 | .87 | .89 | .72 | .74 | .96 | .79 |
| O4 Adventurous | .82 | .79 | .77 | .63 | .66 | .81 | .74 |
| O5 Intellect | .88 | .97 | .70 | .65 | .63 | .93 | .83 |
| O6 Liberalism | 1.12 | 1.11 | .77 | .71 | .73 | .75 | .75 |
| A1 Trust | .82 | .69 | .78 | .63 | .64 | .82 | .75 |
| A2 Morality | .76 | .59 | .66 | .61 | .60 | .89 | .84 |
| A3 Altruism | .57 | .55 | .57 | .47 | .45 | .75 | .67 |
| A4 Cooperation | .85 | .59 | .73 | .62 | .64 | .86 | .79 |
| A5 Modesty | .88 | .77 | .75 | .63 | .62 | .93 | .82 |
| A6 Sympathy | .74 | .69 | .63 | .58 | .62 | .88 | .77 |
| C1 Self-efficacy | .62 | .56 | .60 | .52 | .48 | .78 | .66 |
| C2 Orderliness | .90 | .82 | .80 | .65 | .71 | 1.07 | 1.04 |
| C3 Dutifulness | .64 | .49 | .56 | .48 | .46 | .84 | .70 |
| C4 Achieve-Strive | .66 | .62 | .66 | .56 | .58 | .79 | .75 |
| C5 Self-Discipline | .83 | .88 | .90 | .78 | .79 | .85 | .77 |
| C6 Cautiousness | .91 | .71 | .83 | .69 | .77 | 1.01 | .80 |
| *Note*. Self = Self-report; Informant = Informant-report. | | | | | | | |

Supplementary Table 8.

Correlations among Baseline FFM personality domains and experiential factors

| Measure | 1 | 2 | 3 | 4 | 5 | 6 | 7 | 8 | 9 | 10 | 11 | 12 |
| --- | --- | --- | --- | --- | --- | --- | --- | --- | --- | --- | --- | --- |
| 1. Neuroticism |  |  |  |  |  |  |  |  |  |  |  |  |
| 2. Extraversion | -.58* |  |  |  |  |  |  |  |  |  |  |  |
| 3. Openness | -.07 | .18* |  |  |  |  |  |  |  |  |  |  |
| 4. Agreeableness | -.17 | .09 | .31* |  |  |  |  |  |  |  |  |  |
| 5. Conscientiousness | -.51* | .37* | .03 | .24* |  |  |  |  |  |  |  |  |
| 6. RMEQ Mystical | .03 | .00 | .13 | -.05 | .02 |  |  |  |  |  |  |  |
| 7. RMEQ Positive Mood | .07 | -.01 | .11 | -.03 | .00 | .80* |  |  |  |  |  |  |
| 8. RMEQ Timespace | -.01 | .06 | .13 | .02 | .10 | .65* | .54* |  |  |  |  |  |
| 9. RMEQ Ineffable | .09 | -.09 | .06 | -.01 | -.04 | .50* | .43* | .49* |  |  |  |  |
| 10. Ego Dissolution Inventory | .04 | -.04 | .02 | -.04 | -.10 | .65* | .56* | .57* | .39* |  |  |  |
| 11. AEI Clarity | .03 | .03 | .11 | -.14 | -.02 | .85* | .83* | .55* | .46* | .64* |  |  |
| 12. AEI Reappraisal | .22* | -.12 | .13 | -.07 | -.14 | .64* | .59* | .46* | .41* | .55* | .74* |  |
| 13. AEI Discomfort | .22* | -.19 | .11 | .06 | -.11 | .06 | .03 | .27* | .26* | .11 | .04 | .37* |
| *Note*. RMEQ = Revised Mystical Experience Questionnaire; AEI = Ayahuasca Experience Inventory. | | | | | | | | | | | | |

Supplementary Table 9.

Model fit indices for Ayahuasca Experience Inventory

| Structure | MAP | RMSEA | TLI | SRMR | BIC |
| --- | --- | --- | --- | --- | --- |
| One-factor | .03 | .11 | .44 | .13 | -2846 |
| Two-factor | .02 | .10 | .60 | .06 | -5847 |
| Three-factor | .01 | .09 | .64 | .05 | -6321 |
| *Note.* MAP = Velicer's Minimum Average Partial index; RMSEA = root mean square error of approximation index; TLI = Tucker-Lewis index; SRMR = Standardized Root Mean Square Residual; BIC = Bayesian Information Criterion | | | | | |

Supplementary Table 10.

Exploratory factor analysis item loadings for Ayahuasca Experience Inventory

| Item | Clarity | Reappraisal | Discomfort |
| --- | --- | --- | --- |
| I felt clarity. | **.93** | -.11 |  |
| I felt centered | **.92** |  |  |
| I experienced profound inner peace. | **.84** |  | -.18 |
| I basked in my experience of the ceremony | **.81** |  |  |
| I experienced a kind of awe. | **.81** |  | .12 |
| I experienced an all embracing love. | **.80** |  |  |
| I was able to perceive my thoughts/feelings with exceeding clarity. | **.80** |  |  |
| I saw a roadmap for living in alignment with my authentic self | **.80** |  |  |
| I felt extraordinary powers within myself. | **.79** |  |  |
| I felt the sentiment, "I am on the right path." | **.79** | -.11 |  |
| I felt connected to myself | **.77** |  | -.14 |
| My purpose in life became clear | **.77** | -.11 |  |
| I experienced boundless pleasure. | **.74** |  |  |
| I had insights into connections that had previously puzzled me. | **.74** |  | .15 |
| Many things appeared to me as breathtakingly beautiful. | **.71** |  |  |
| I had very original thoughts. | **.71** | -.16 | .15 |
| I felt extreme love and acceptance for myself. | **.64** | .19 |  |
| I felt great trust in myself. | **.63** | .18 | -.14 |
| I felt totally free and released from all obligations. | **.58** |  |  |
| I was able to understand the meaning of life. | **.56** | .18 |  |
| Bodily sensations were very enjoyable. | **.50** |  |  |
| I felt like I was able to accept everything for what it was | **.49** | .27 | -.14 |
| Conflicts and contradictions seemed to dissolve. | **.49** | .26 |  |
| Worries and anxieties of everyday life felt unimportant. | **.47** | .18 |  |
| I realized how much I value myself | **.46** | .38 | -.11 |
| Felt trusting of others in the room. | **.43** | .18 |  |
| Things in my environment had a new strange meaning. | **.42** | .26 |  |
| I felt boundless love for people close to me. | **.40** | .36 |  |
| I felt connected to others in the ceremonial space | **.36** | .24 |  |
| I could see images from my memory or imagination with extreme clarity. | **.35** | .29 |  |
| Many things seemed incredibly funny to me. | **.33** | .26 |  |
| Felt a desire to repair a relationship | **.27** | .23 |  |
| Felt gratitude for the lessons I learned from people that had wronged me | -.17 | **.90** | -.16 |
| Felt gratitude for the little or previously insignificant moments in my life |  | **.85** | -.16 |
| I considered that I am too hard on myself. | -.15 | **.76** |  |
| I realized that I am unncessarily bound to repeat patterns of acting/feeling/behaving | -.18 | **.74** |  |
| I was able to see new positive meaning in a past trauma |  | **.73** |  |
| Laughter or humor helped me overcome conflicts, fears, or difficult past experiences. |  | **.72** |  |
| Felt gratitude for the challenges life had given me |  | **.71** | -.11 |
| I saw the humor in situations that I previously may have taken too seriously |  | **.71** | -.14 |
| I felt that I was forced to confront negative perceptions I've had of myself | -.13 | **.71** | .22 |
| I recognized that I can live more freely, without a sense of obligation. |  | **.70** | -.15 |
| I identified aspects of myself that cause me pain. |  | **.69** | .11 |
| I realized that I am unnecessarily bound to follow social norms |  | **.65** |  |
| I realized how courageous I am. |  | **.64** |  |
| I felt forgiveness | .18 | **.59** |  |
| I felt deep compassion for people who have wronged me or people close to me |  | **.55** |  |
| I felt courageous | .16 | **.54** |  |
| I related to my physical pain with a sense of braveness |  | **.52** |  |
| I felt capable of living courageously | .31 | **.50** |  |
| I realized that I can just be, without obligation or pressure to act in a particular way | .31 | **.50** | -.13 |
| I realized how critical it is that I make changes in my life. | .18 | **.50** | .11 |
| I faced my fears | .24 | **.42** | .27 |
| I overcame/resolved my fears | .40 | **.40** |  |
| I wrestled with my inner conflicts | .20 | **.39** | .36 |
| Felt gratitude for the love and support of those around me | .31 | **.36** | .11 |
| Felt a disappearance of opposing forces such as good and bad, right and wrong. | .16 | **.36** |  |
| I felt that I have great potential in me to achieve what I need in life. | .35 | **.34** |  |
| Engaged in problem-solving about how to communicate with someone. | .26 | **.34** |  |
| I experienced deep compassion for others | .30 | **.33** |  |
| I felt great compassion for others' distress or pain. | .31 | **.31** |  |
| I felt that I missed loved ones greatly. | .23 | **.26** | .12 |
| I felt tormented. |  | -.15 | **.88** |
| I experienced everything as frighteningly distorted. |  |  | **.82** |
| I felt threatened. |  | -.18 | **.82** |
| I felt lost | -.11 |  | **.81** |
| I felt overwhelmed by the experience | .11 | -.21 | **.79** |
| I felt as if dark forces had overtaken me. |  | -.12 | **.78** |
| I felt an unpleasant flow of information that I couldn't control |  |  | **.75** |
| Time passed slowly in a tormenting way. | -.12 |  | **.74** |
| I felt isolated from everything and everyone. |  | -.16 | **.74** |
| I felt agony. |  | .16 | **.73** |
| I was afraid that the state I was in would last forever. |  |  | **.68** |
| I experienced my surroundings as strange and weird. |  |  | **.64** |
| I experienced great physical discomfort | -.29 | .32 | **.55** |
| I felt exhausted. |  | .16 | **.51** |
| I stayed frozen in an very unnatural position for an extended period of time. | .12 |  | **.46** |
| *Note*. Loadings with absolute values less than .1 were removed. Strongest loading for each item is in bold. | | | |

Supplementary Table 11.

Shamanic elements

| Item name | Item Content |
| --- | --- |
| Trusted shaman | I trusted the shaman(s) |
| Mesmerized icaro | I was mesmerized by the icaro's song |
| Icaro healing | I felt that the icaro prayer was healing parts of me |
| Medicine cleaning | I felt that the medicine was cleaning me. |
| Struggled to purge | I struggled to purge. |
| War with entity | As I attempted to purge, I felt at war with some external entity who was responsible. |
| Purging self | I felt that I was purging out an aspect of myself (e.g., addiction, depression). |
| Purged physical ailment | I purged a physical issue or ailment. |
| Purged completely | I felt that I purged completely |
| Viewed object | I saw what someone else purged. |
| Relationship object | I saw that what someone else purged was related to something that I had experienced as well. |
|  |  |

**Supplementary Figures**


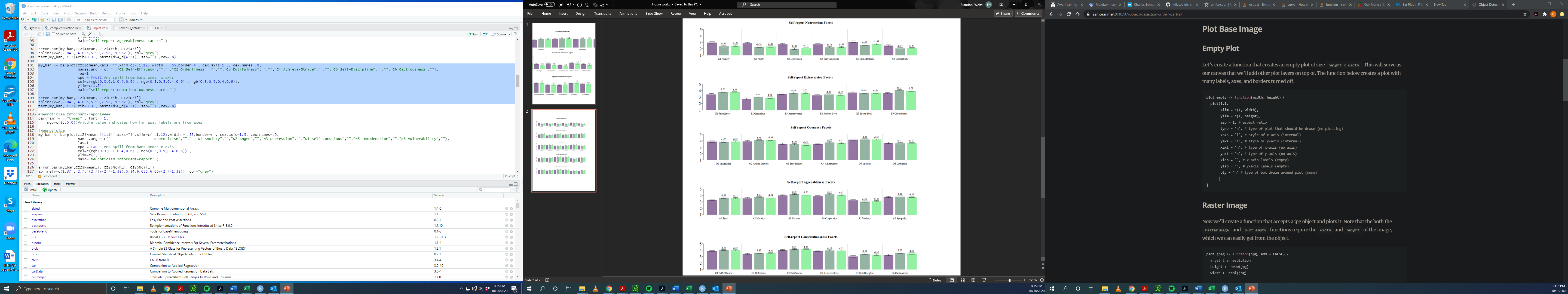


**Supplementary Figure 1.** Box plots illustrating self-reported change in personality facets. Cohen’s *dz* values indicate effect size change from Baseline.
